# Common DNA methylation changes in biliary tract cancers identify subtypes with different immune characteristics and clinical outcomes

**DOI:** 10.1186/s12916-021-02197-w

**Published:** 2022-02-07

**Authors:** Zhiquan Qiu, Jun Ji, Yu Xu, Yan Zhu, Chunfang Gao, Guoqiang Wang, Chengcheng Li, Yuzi Zhang, Jing Zhao, Chenyang Wang, Xiaofang Wen, Zhou Zhang, Bingsi Li, Zhihong Zhang, Shangli Cai, Bin Li, Xiaoqing Jiang

**Affiliations:** 1 Department of Biliary Tract Surgery I, Eastern Hepatobiliary Surgery Hospital, Secondary Military Medicine University, No. 225 Changhai Road, Shanghai, 200438 China; 2 Department of Laboratory Medicine, Eastern Hepatobiliary Surgery Hospital, Secondary Military Medicine University, Shanghai, China; 3 Department of Medicine, Burning Rock Biotech, Guangzhou, China; 4 Department of Pathology, Changhai Hospital, Shanghai, China; 5 Department of Research and Development, Burning Rock Biotech, Guangzhou, China; 6 Department of Bioinformatics, Burning Rock Biotech, Guangzhou, China

**Keywords:** Biliary tract cancer, DNA methylation, Prognostication, Immune characteristic

## Abstract

**Background:**

DNA methylation-associated studies on biliary tract cancer (BTC), including cholangiocarcinoma (CCA) and gallbladder cancer (GBC), may improve the BTC classification scheme. We proposed to identify the shared methylation changes of BTCs and investigate their associations with genomic aberrations, immune characteristics, and survival outcomes.

**Methods:**

Multi-dimensional data concerning mutation, DNA methylation, immune-related features, and clinical data of 57 CCAs and 48 GBCs from Eastern Hepatobiliary Surgery Hospital (EHSH) and 36 CCAs in the TCGA-CHOL cohort were analyzed.

**Results:**

In our cohort including 24 intrahepatic CCAs (iCCAs), 20 perihilar CCAs (pCCAs), 13 distal CCAs (dCCAs), and 48 GBCs, 3369 common differentially methylated regions (DMRs) were identified by comparing tumor and non-tumor samples. A lower level of methylation changes of these common DMRs was associated with fewer copy number variations, fewer mutational burden, and remarkably longer overall survival (OS, hazard ratio [HR] = 0.07, 95% confidence interval [CI] 0.01–0.65, *P* = 0.017). Additionally, a 12-marker model was developed and validated for prognostication after curative surgery (HR = 0.21, 95% CI 0.10–0.43, *P* < 0.001), which exhibited undifferentiated prognostic effects in subgroups defined by anatomic location (iCCAs, d/pCCAs, GBCs), TNM stage, and tumor purity. Its prognostic utility remained significant in multivariable analysis (HR = 0.26, 95% CI 0.11–0.59, *P* = 0.001). Moreover, the BTCs with minimal methylation changes exhibited higher immune-related signatures, infiltration of CD8^+^ lymphocytes, and programmed death-ligand 1 (PD-L1) expression, indicating an inflamed tumor immune microenvironment (TIME) with PD-L1 expression elicited by immune attack, potentially suggesting better immunotherapy efficacy.

**Conclusions:**

In BTCs, DNA methylation is a powerful tool for molecular classification, serving as a robust indicator of genomic aberrations, survival outcomes, and tumor immune microenvironment. Our integrative analysis provides insights into the prognostication after curative surgery and patient selection for immunotherapy.

**Supplementary Information:**

The online version contains supplementary material available at 10.1186/s12916-021-02197-w.

## Background

Biliary tract cancers (BTCs), including cholangiocarcinoma (CCA) and gallbladder cancer (GBC), are rare but aggressive [1]. Multi-omics analysis may improve the previous classification framework based on anatomic location and pathological features and provide insight into the mechanism of tumorigenesis and potential targets for precision medicine.

BTCs at different anatomic locations may display similar genomic/epigenomic features. Previous comparative exome sequencing studies have found commonalities among BTCs in different locations, such as *TP53*, *KRAS*, *KMT2C*, and *SMAD4* mutations, with a second tier of less frequently mutated genes including *ARID1A*, *CDKN2A*, and *PIK3CA* [2–10]. Mutational differences between CCAs and GBCs have tended to be in the frequency of mutations in certain genes, rather than different pathways of genes being mutated [2, 5]. As for DNA methylation, the DNA methylation pattern of CCA and its association with prognosis have been reported in several studies [8–11]. As for GBC, the gradual methylation changes in the sequence of gallstone disease, dysplasia, and gallbladder cancer have been described [12], indicating the importance of DNA methylation in GBC carcinogenesis. There are several studies illustrating the associations of survival with the methylation of specific genes in GBCs, e.g., *WIF1* and *WISPIN* [13–15], while the prognostic effect of DNA methylation changes has not been not fully addressed. In addition, no comparative study to date has identified the similarities among BTC methylomes and further explores their associations with prognosis and potential benefit from precision medicine.

Here, we identified 3369 common differentially methylated regions (DMRs) between CCAs and GBCs and uncovered their associations with genomic aberration, survival outcome, and immune characteristics. Of note, the BTCs with minimal methylation changes exhibited an inflamed tumor immune microenvironment (TIME) with programmed death-ligand 1 (PD-L1) expression elicited by immune attack, potentially suggesting better immunotherapy efficacy.

## Methods

### Patients

The included samples of the Eastern Hepatobiliary Surgery Hospital (EHSH) cohort consist of three parts: cancerous, adjacent, and precancerous tissues. The 105 tumor samples (24 intrahepatic CCAs [iCCAs], 20 perihilar CCAs [pCCAs], 13 distal CCAs, and 48 GBCs) and 50 adjacent non-tumor samples (gallbladder [*n* = 28] and bile duct [*n* = 22]) were obtained immediately following surgery at EHSH from October 2017 to September 2019. The diagnosis and tumor purity were confirmed by two independent pathologists based on the resected samples. Characteristics of the cancerous and adjacent normal tissues are shown in Additional file 1: Table S1-2, respectively. All participants had not received anti-tumor treatment before surgery. Participants with carcinoma or benign disease of other organs were excluded from this study. In addition, eight samples of precancerous disease (e.g., gallbladder polyps) were collected for exploratory analysis (Additional file 1: Table S3).

The Cancer Genome Atlas-cholangiocarcinoma (TCGA-CHOL) cohort of 36 patients with epigenomic, transcriptomic, mutational, immune-related, and survival (OS and progression-free interval [PFI]) data were analyzed in the present study [10, 16]. The immune characteristics of this cohort (e.g., signatures and immune cell sorting) were retrieved from the study of Thorsson et al. [16].

Patients or the public were not involved in the design, conduct, reporting, or dissemination plans of our research. All collection and usage of human samples and clinical data were in accordance with the principles of the Declaration of Helsinki and approved by the Ethics Committee of Eastern Hepatobiliary Surgery Hospital (EHBHKY2018-02-014). The written consents were received from all the participated patients. This report follows the Strengthening the Reporting of Observational Studies in Epidemiology (STROBE) reporting guideline.

### DNA methylation profiling and identification of differentially methylated region (DMR)

All sequencing experiments were implemented in a College of American Pathologists (CAP)- and Clinical Laboratory Improvement Amendments (CLIA)-certified laboratory (Burning Rock Biotech, Guangzhou, China) before May 2020. The procedure for DNA extraction was as previously described [17]. In brief, DNA was extracted with a QIAamp DNA formalin-fixed paraffin-embedded (FFPE) tissue kit according to the manufacturer’s instructions. DNA concentration was measured by the Qubit double-stranded DNA assay (Life Technologies, Carlsbad, CA, USA).

As for methylation sequencing, a capture-based method, SeqCap Epi CpGiant Probes (Roche Sequencing Solutions, Madison, WI, USA) was performed to detect > 5.5 million CpG sites (capture size, 80.5 Mb) [18]. We generated a bisulfite sequencing library with the brELSA™ method (Burning Rock Biotech, Guangzhou, China) [19]. The target libraries were quantified by real-time PCR and sequenced on NovaSeq 6000 with 50× target depth on average.

Since differentially methylated regions consisting of multiple CpG sites played more important roles than a single CpG site in cancer detection as reported [20], we defined 319,133 methylation regions of CpG sites with close genomic distance and highly correlation in methylation level [19], which were analyzed in the present study.

### NGS testing and analysis of mutation and copy number variation

As for mutation sequencing, a capture-based targeted deep sequencing was performed using a 520-gene panel, spanning 1.64 Mb of the human genome (included genes are shown in Additional file 1: Table S4). Detailed descriptions of sequencing and capturing single nucleotide variant and copy number variation are shown in Additional file 2: Method S1.

Mutated genes included in our analysis were restricted to non-silent mutations consisting of non-sense mutation, missense mutation, frameshift mutation, inframe mutation, splice site mutation, translation start site mutation, and non-stop mutation. Truncating mutations of oncogene were excluded because most of these are passenger mutations with limited cancer-promoting function.

The signaling pathways and their members we analyzed are shown in Additional file 1: Table S5. The definition of pathways drew upon previous genomic studies [3, 5].

### Assessment of programmed death-ligand 1 (PD-L1) protein expression

For each tumor FFPE block, a 5-μm section was cut and stained with the Dako 22C3 mouse monoclonal antibody with Dako Autostainer Link-48 platform according to the manufacturer’s instructions. Cores showing a neoplastic component ≥ 30% were considered as adequate. PD-L1 positivity was determined by any expression in tumor cells or immune cells evaluated independently by two pathologists.

### Statistical analysis

To assess the between-group difference, we performed the (i) Fisher exact test, chi-square test, and Cochran-Armitage test for trend for categorical variables; (ii) Mann-Whitney test for continuous variables; and (iii) Kaplan-Meier (KM) method, log-rank method, and Cox regression (hazard ratio [HR] and 95% confidence interval [CI]) for survival variables (OS and PFI). The covariates with *P* value below 0.05 in the univariable analysis were included in the following multivariable model. The differentiated methylation regions (DMRs) between tumors and adjacent tissues were selected by the Mann-Whitney test for further clustering. Detailed description of single sample gene set enrichment analysis is shown in Additional file 2: Method S2.

The non-supervised clustering of methylation data in both the EHSH and TCGA cohorts was performed by the *K*-means method (R package, ConsensusClusterPlus). Gene Ontology (GO) enrichment analysis was performed by R (R package, clusterProfiler) [21–23]. The 12-DMR prognostic model was built via least absolute shrinkage and selection operator (LASSO).

All statistical analyses mentioned above were performed using IBM SPSS Statistics 22 and R 3.4.2, and the graphs were drawn by GraphPad Prism 9 and R 3.4.2. The nominal level of significance was set as 5%, and all 95% CIs were 2-sided.

## Results

### Identification of common DMRs among BTCs

**Fig. 1:**
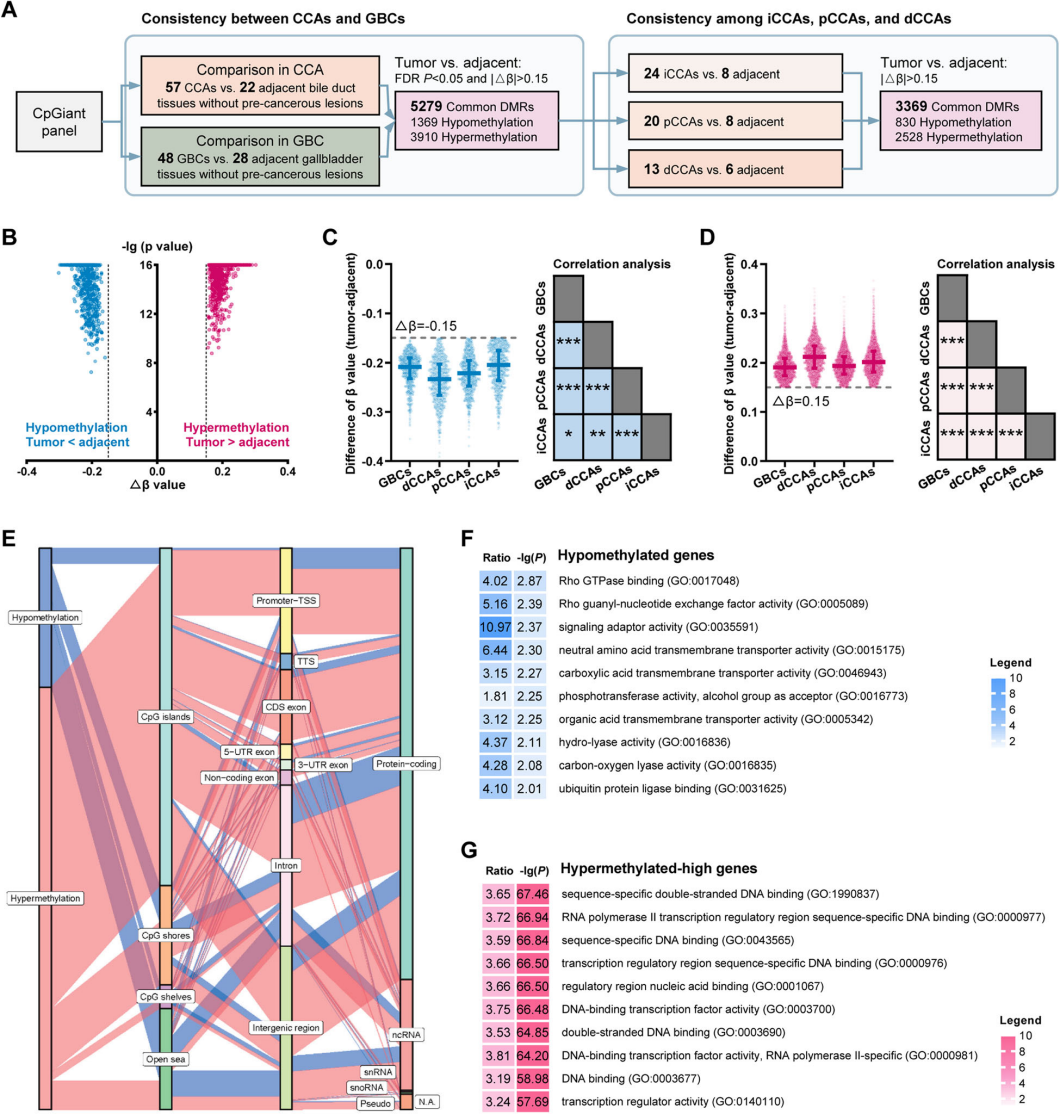
Identification of the common DMRs among BTCs in the EHSH cohort. **A** Diagram of the identification process. **B** Volcano plot illustrating the *P* value and *β* value difference of the 3369 DMRs. **C** The *β* value difference of the hypomethylation DMRs and their inner-correlations among iCCAs, pCCAs, dCCAs, and GBCs. **D** The *β* value difference of the hypermethylation DMRs and their inner-correlations among iCCAs, pCCAs, dCCAs, and GBCs. **E** Sankey plot of the hypomethylation and the hypermethylation DMRs. **F**, **G** Gene Ontology enrichment analyses of the hypomethylation- (**F**) and the hypermethylation-associated genes (**G**). BTC, biliary tract cancer; i/p/dCCA, intrahepatic/perihilar/distal cholangiocarcinoma; DMR, differentially methylated region; GBC, gallbladder cancer; GO, Gene Ontology

By comparing the methylomes of cancerous and normal adjacent tissues separately in CCAs (57 treatment-naïve CCAs vs. 22 adjacent bile ducts) and GBCs (48 treatment-naïve GBCs vs. 28 adjacent gallbladders), 5279 significant DMRs with *β* value difference above 0.15 in both CCAs and GBCs were identified (Fig. 1A). Moreover, we assessed the consistency of these 5279 DMRs among iCCAs, pCCAs, and dCCAs, and 3369 DMRs had minimal *β* value differences of 0.15 in all three subgroups (Fig. 1A). Of these, 835 DMRs showed lower *β* values in tumor samples compared to adjacent non-tumor samples (referred to as hypomethylation DMRs), and the other 2534 DMRs exhibiting higher *β* values were referred to as hypermethylation DMRs. All of the 3369 DMRs showed great significance by comparing BTCs and adjacent non-tumor samples (maximal *P* value = 5.66 × 10^−8^, FDR *P* value< 0.05, Fig. 1B). In addition, we found significant positive correlations of the *β* value differences of the 835 hypomethylation DMRs among iCCAs, pCCAs, dCCAs, and GBCs (Fig. 1C). Similarly, significant positive correlations were found in the 2534 hypermethylation DMRs (Fig. 1D). These correlations further indicate the consistency of these DMRs among the BTCs at different anatomic locations.

The hypermethylation DMRs were enriched in the regions of CpG islands, promoters, and exons of coding sequence, but not CpG shores, CpG shelves, open sea, introns, and intergenic regions (Fig. 1E and Additional file 1: Table S6). The top 50 hypermethylation and hypomethylation DMRs are displayed in Additional file 1: Table S7 and S8, respectively. GO analysis revealed that hypomethylation mainly affected the genes related to Rho GTPase binding, transmembrane transporter activity, and lyase activity (Fig. 1F), and hypermethylation targeted DNA binding and transcription (Fig. 1G).

### Prognostic correlates of DNA methylation-based clusters

**Fig. 2:**
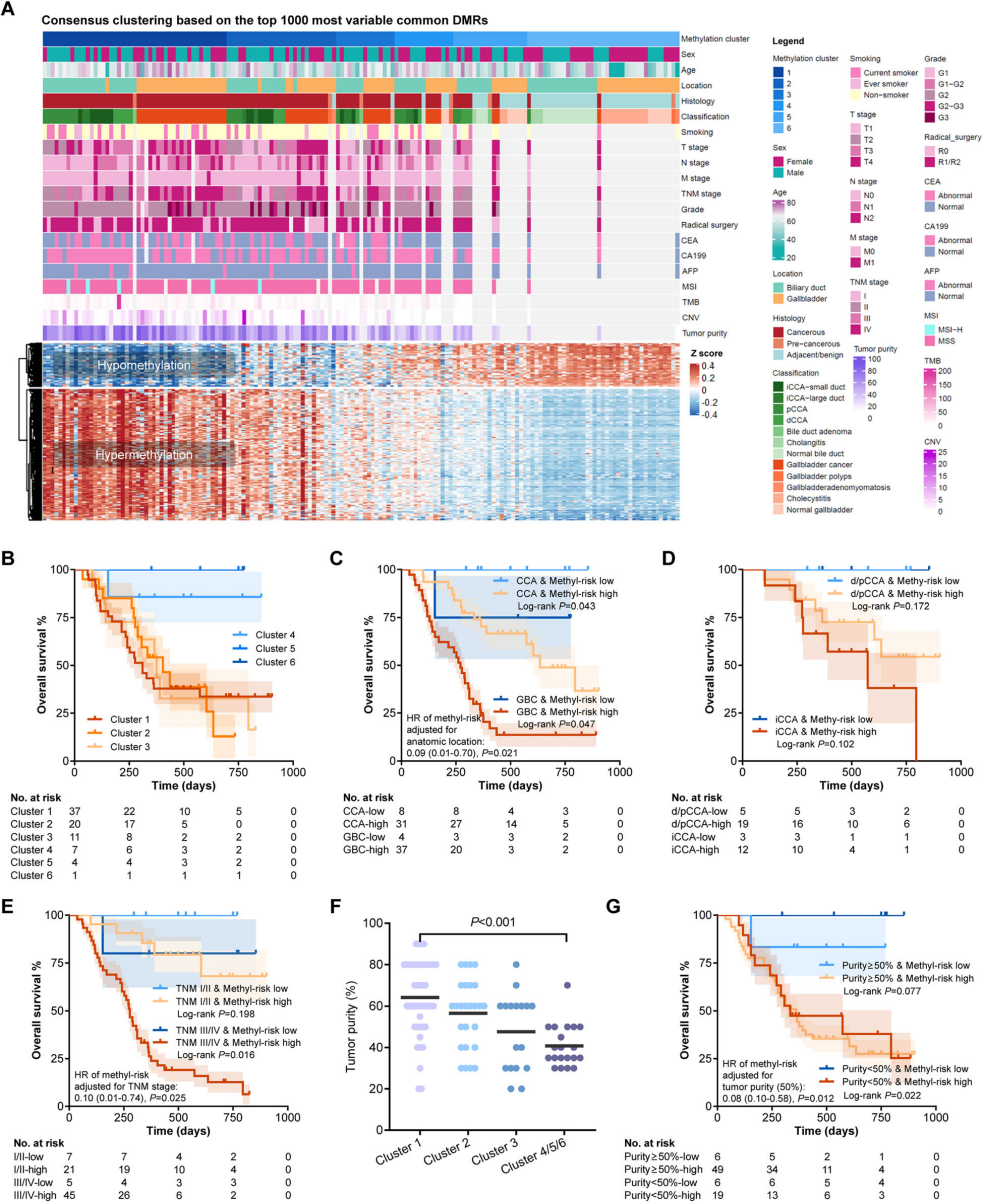
Association between global methylation changes and prognosis in the EHSH cohort. **A** Clinicopathological and methylation data of 163 samples (105 treatment-naïve BTC samples, 50 adjacent non-precancerous bile duct or gallbladder tissues, and 8 precancerous lesions). **B**–**E** Kaplan-Meier curves illustrating the OS data of 80 BTC patients of the six methylation-based clusters (**B**), the subgroups classified by cluster-based risk and anatomic location (**C**, **D**), and the subgroups classified by cluster-based risk and TNM stage (**E**). **F** Tumor purity of the samples in different clusters. **G** Kaplan-Meier curves illustrating the OS data of 80 cancer patients of the subgroups classified by cluster-based risk and tumor purity. BTC, biliary tract cancer; CCA, cholangiocarcinoma; GBC, gallbladder cancer; OS, overall survival

Using the top 1000 most variable DMRs of the 3369 DMRs identified above, 163 tissues (105 treatment-naïve BTC samples, 50 adjacent non-precancerous bile duct or gallbladder tissues, and 8 precancerous lesions) were classified into 6 groups by non-supervised consensus clustering (Fig. S1). Baseline characteristics of the abovementioned samples and the comparison among iCCAs, d/pCCAs, and GBCs are shown in Additional file 1: Table S1-3. As delineated in the heatmap (Fig. 2A), ranging from the methylation cluster 1 to cluster 6, the degree of global methylation changes gradually decreased, and the proportion of non-precancerous adjacent tissues gradually increased. Despite that the GBCs in our cohort exhibited poorer histological grade and later TNM stage compared to iCCAs and d/pCCAs (Additional file 1: Table S1), similar proportions of GBCs, dCCAs, pCCAs, small-duct iCCAs, and large-duct iCCAs were observed in different methylation clusters (*P* = 0.72), indicating that the methylation clusters based on the 3369 common DMRs were independent of anatomic sites and potentially reflected a shared characteristic among the cancers along biliary tract.

As non-precancerous adjacent tissues were enriched in the methylation clusters 4–6 with minimal methylation changes (Fig. 2A), we speculated that the BTCs in clusters 4–6 might exhibit lower invasiveness, and therefore, clusters 4–6 might be associated with longer survival. Among the 80 BTC patients with OS data (characteristics are shown in Additional file 1: Table S1), clusters 4–6 showed better prognosis compared to clusters 1–3 (HR = 0.07, 95% CI 0.01–0.65, *P* = 0.017, Fig. 2B). Similar trends were observed in the subgroups classified by anatomic location, i.e., iCCAs, d/pCCAs, and GBCs (Fig. 2C, D), and stage, i.e., stages I–II and stage III–IV (Fig. 2E). We hereby defined the cluster-based risk according to our methylation clusters (high cluster-based risk, clusters 1–3; low cluster-based risk, clusters 4–6). We further use the top 500 and 250 most variable DMRs to perform non-supervised clustering. Compared to the one using top 1000 DMRs as described above, 95.7% of the samples had the same results of the cluster-based risk (Additional file 1: Table S9), indicating the robustness of the classification by common methylation DMRs among BTCs.

Tumor purity (also termed as tumor cellularity) might somewhat influence the sequencing data of methylome and therefore the clustering outcome. Similar to the result of Goeppert et al.’s study in iCCAs, we observed slightly lower tumor cellularity in the samples with lower methylation changes (average, 40.8% in clusters 4–6 compared to 64.1%, 56.5%, and 47.7% in clusters 1, 2, and 3, respectively, Fig. 2F). Lower tumor purity might indicate a higher proportion of stromal area and/or higher density of tumor-infiltrating immune cells, which was partially suggested by the lower *CD8A* methylation in clusters 4–6 which might reflect higher infiltration of CD8^+^ T cells. Despite the slight difference of tumor purity in different clusters, tumor purity was not linked with OS (continuous variable: HR = 1.00, 95% CI 0.99–1.02, *P* = 0.933; categorical variable [≥ 50% vs. < 50%]: HR = 1.00, 95% 0.99–1.02, *P* = 0.672). The cluster-based risk showed an undifferentiated prognostic effect in the patients with higher tumor purity (≥ 50%, *P* = 0.077) and lower tumor purity (< 50%, *P* = 0.022, Fig. 2G). These results rule out the influence of tumor purity on the prognostic effect of the cluster-based risk.

To investigate whether the prognostic effect of the cluster-based risk is independent of other variables, we firstly analyzed the association between clusters and key clinicopathological variables, including sex, age, smoking history, anatomic location, TNM stage, histological grade, resection margin, carcinoembryonic antigen (CEA), carbohydrate antigen 19-9 (CA19-9), alpha-fetoprotein (AFP), and multiple immunohistochemical staining markers (e.g., Hep-1, Mucin 1 [MUC-1], P63, and S100). No significant association was revealed except the lower frequency of CEA abnormality in clusters 4–6 (*P* = 0.007, Additional file 1: Table S10). Moreover, univariable analyses discovered that anatomic location, TNM stage, histological grade, resection margin, and CEA abnormality were significantly associated with OS in addition to the cluster-based risk (Table 1). In the multivariable model, OS was significantly associated with the cluster-based risk (HR = 0.13, 95% CI 0.02–0.96, *P* = 0.045), rather than anatomic site (Table 1). These results suggest that the methylation-based clusters might be an independent biomarker predicting prognosis in BTCs.

**Table 1:** Univariable and multivariable analyses of OS in the EHSH cohort

Parameter	Univariable analysis		Multivariable analysis 1 (cluster-based risk)		Multivariable analysis 2 (LASSO-based risk)	
	HR (95% CI)	*P* value	HR (95% CI)	*P* value	HR (95% CI)	*P* value
Age (≥ 65 vs. < 65)	0.67 (0.37–1.23)	0.20				
Sex (male vs. female)	0.73 (0.41–1.31)	0.29				
Anatomic site		< 0.001		0.70		0.98
Extrahepatic CCA vs. GBC	0.22 (0.10–0.49)	< 0.001	0.66 (0.22–1.93)	0.45	1.10 (0.36–3.34)	0.87
Intrahepatic CCA vs. GBC	0.38 (0.17–0.86)	0.021	0.72 (0.26–1.96)	0.52	1.08 (0.39–3.03)	0.88
GBC (dummy variable)						
Smoking (smoker vs. non-smoker)	1.24 (0.67–2.31)	0.49				
TNM stage (III/IV vs. I/II)	5.55 (2.96–10.41)	< 0.001	3.92 (1.22–12.59)	0.021	3.80 (1.17–12.39)	0.027
Resection margin (R0 vs. R1/R2)	0.28 (0.15–0.53)	< 0.001	0.48 (0.24–0.96)	0.038	0.43 (0.22–0.86)	0.016
Histological grade (> G2 vs. ≤ G2)	2.86 (1.50–5.46)	0.001	1.81 (0.84–3.90)	0.13	1.84 (0.85–4.00)	0.12
Microsatellite (unstable vs. stable)	1.65 (0.40–6.86)	0.49				
CEA (ng/mL, > 5 vs. ≤ 5)	1.84 (1.02–3.30)	0.042	1.08 (0.53–2.18)	0.84	1.38 (0.70–2.73)	0.35
CA199 (U/mL, > 40 vs. ≤ 40)	0.74 (0.40–1.37)	0.33				
AFP (ng/mL, > 20 vs. ≤ 20)	0.30 (0.04–2.18)	0.23				
Tumor purity (continuous variable)	1.00 (0.99–1.02)	0.93				
Cluster-based risk (low vs. high)	0.09 (0.01–0.65)	0.017	0.13 (0.02–0.96)	0.045		
LASSO-based risk (low vs. high)	0.21 (0.10–0.43)	< 0.001			0.26 (0.11–0.59)	0.001

*AFP* alpha-fetoprotein, *CA199* carbohydrate antigen 199, *CCA* cholangiocarcinoma, *CEA* carcinoembryonic antigen, *GBC* gallbladder cancer, *NA* not applicable

### Prognostic correlates of the methylation level of individual DMRs.

**Fig. 3:**
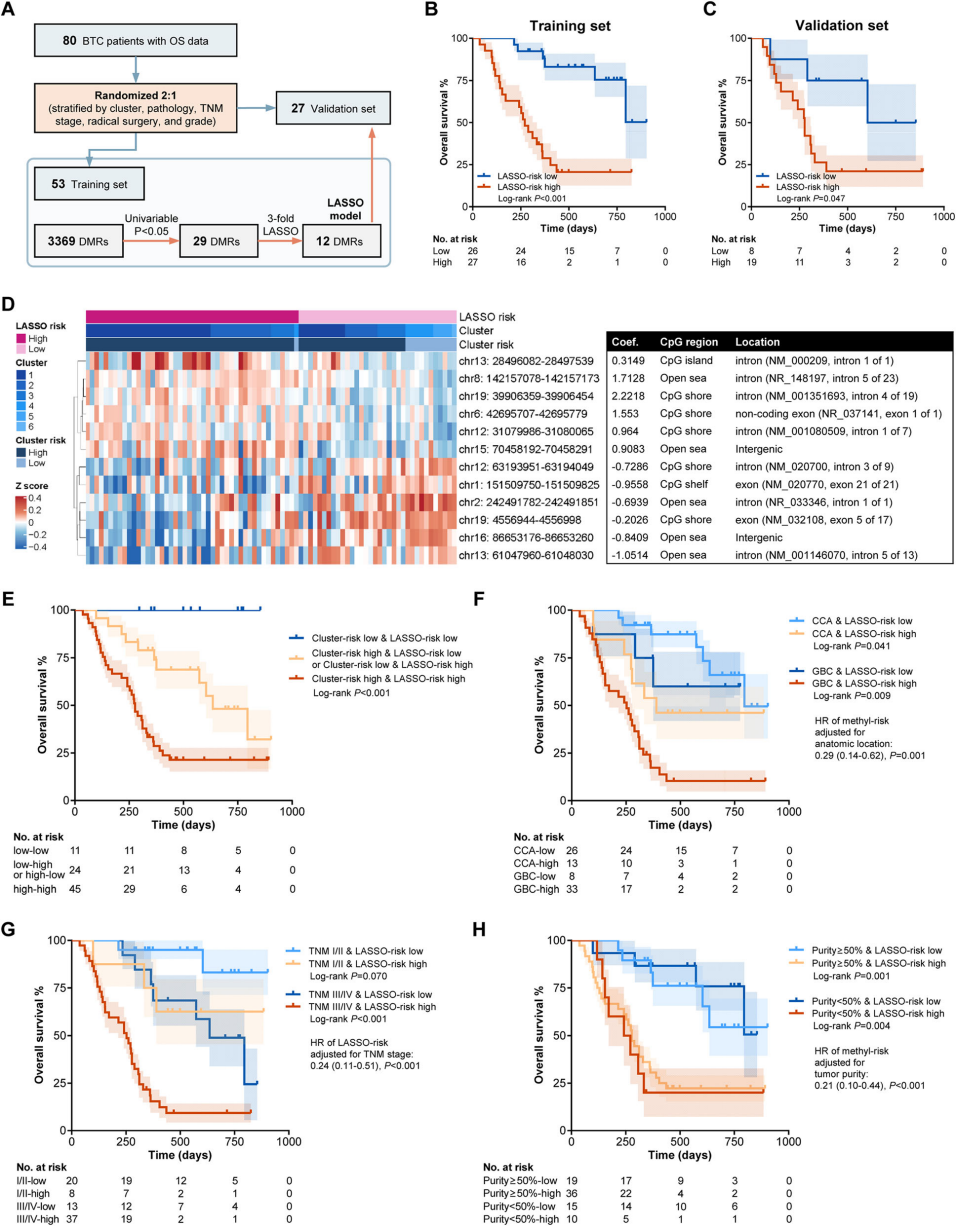
Association between individual methylation DMRs and prognosis in the EHSH cohort. **A** Diagram of the workflow of developing the 12-DMR model. **B**, **C** Association between the LASSO-based risk and overall survival in the training set (**B**) and the validation set (**C**). **D** Heatmap and table illustrating the methylation data and features of the 12 DMRs involved in the LASSO model. **E**, **F** Kaplan-Meier curves illustrating the OS data of 80 BTC patients of the subgroups classified by LASSO-based risk and TNM stage (**E**) and the subgroups classified by LASSO-based risk and anatomic location (**F**). BTC, biliary tract cancer; DMR, differentially methylated region; LASSO, least absolute shrinkage and selection operator

To specifically discover the prognosis-related DMRs, 80 BTC patients with OS data were randomly assigned to the training and validation sets with a 2:1 ratio (Fig. 3A), and univariable analysis revealed 54 prognosis-related DMRs in the training set. Based on these 54 DMRs, LASSO regression was performed and constructed a LASSO score based on 12 DMRs (optimal lambda selection and LASSO coefficient profiles are shown in Fig. S2 and Additional file 1: Table S11, respectively). Using this score and the cutoff (median value) derived from the training set, we observed a consistent prognostic effect in both the training set (*P* < 0.001, Fig. 3B) and the validation set (*P* = 0.047, Fig. 3C). In the total 80 BTC patients (methylation data are shown in Fig. 3D), a lower LASSO-based risk was associated with better OS (HR = 0.21, 95% CI 0.10–0.43, *P* < 0.001, Table 1). Combining the LASSO-based risk and the cluster-based risk could differ the BTC patients into subgroups with distinct survivals (*P* < 0.001, Fig. 3E). The prognostic effect of the LASSO-based risk was undifferentiated in the subgroups classified by anatomic location (GBC: *P* = 0.009; CCA: *P* = 0.041, Fig. 3F), TNM stage (stages I–II: *P* = 0.070; stages III–IV: *P* < 0.001, Fig. 3G), or tumor purity (≥ 50%: *P* = 0.001; < 50%: *P* = 0.004). Furthermore, in the multivariable model, the LASSO-based risk (HR = 0.26, 95% CI 0.11–0.59, *P* = 0.001), rather than the anatomic site (d/pCCA vs. iCCA vs. GBC), was associated with OS (Table 1). These results indicate the good robustness of LASSO-based risk that may be effective regardless of other key variables including TNM stage and anatomic location.

### Genomic correlates of DNA methylation-based clusters

**Fig. 4:**
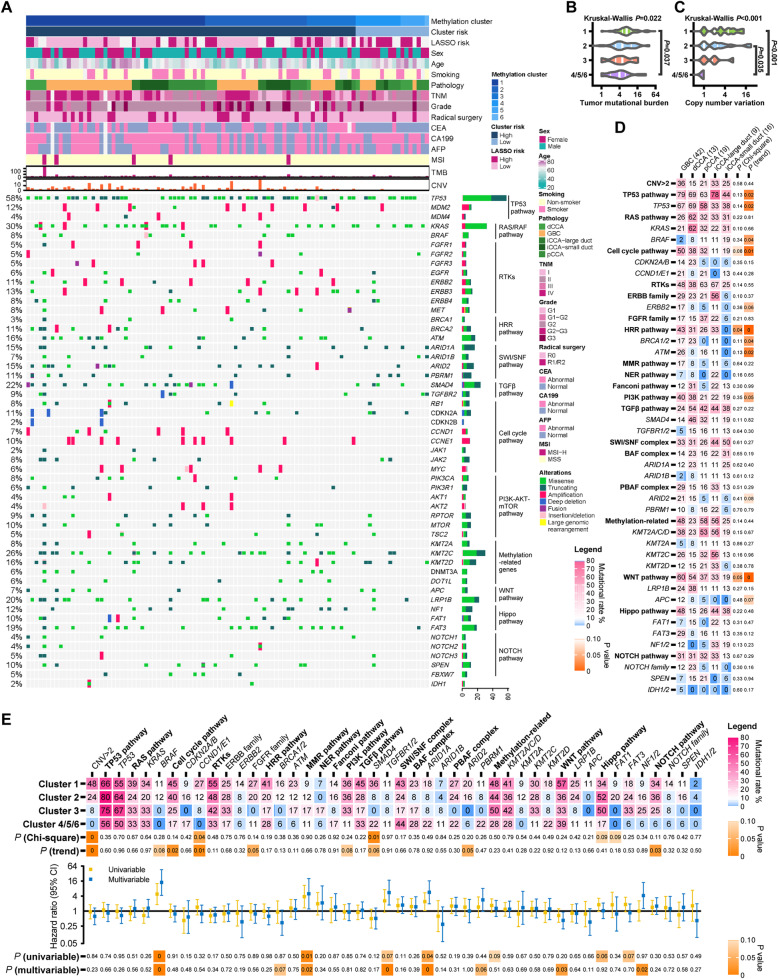
Association between methylation cluster and mutational events in the EHSH cohort. **A** Clinicopathological and mutational data of the 99 BTC samples. **B**, **C** Associations of the methylation-based clusters with TMB (**B**) and CNV (**C**). **D** Association between anatomic location and mutational rate. **E** Associations of mutation with the methylation-based clusters and overall survival. BTC, biliary tract cancer; CCA, cholangiocarcinoma; CNV, copy number variation; DMR, differentially methylated region; GBC, gallbladder cancer; LASSO, least absolute shrinkage and selection operator; TMB, tumor mutational burden

Of the 105 tumor samples, 99 received targeted deep sequencing of mutational events of the coding sequence and splice sites, large genomic rearrangement, copy number variation (CNV), fusion, and microsatellite (Fig. 4A). Tumor mutational burden (TMB) was slightly higher in cluster 1 (Fig. 4B), and CNV events were enriched in clusters 1–2 (Fig. 4C), indicating the association between methylation changes and chromatin instability.

The mutational frequencies of *TP53*, *BRCA1/2*, *ATM*, and PI3K; WNT; homologous recombination repair (HRR); and cell cycle pathway gradually decreased with the order of the anatomical position (GBC, dCCA, pCCA, iCCA-large duct, and iCCA-small duct), while the changing trend of *BRAF* mutational frequency was the opposite (Fig. 4D). Similar differences were observed between the GBCs and the CCAs of the public cohorts in cBioPortal [3–7, 24, 25]. In addition, *CCND1/E*1 and *APC* mutations were enriched in metastatic BTCs (Fig. S3).

Ranging from clusters 1, 2, and 3 to 4–6, the mutational frequency of cell cycle pathway (*P* = 0.022), *CCND/E1* (*P* = 0.005), FGFR family (*P* = 0.049), and NOTCH pathway (*P* = 0.029) decreased gradually (Fig. 4E), suggesting the potential association between these mutational events and DNA methylation changes. In addition, mutations in the MMR pathway, *BRAF*, *TGFBR1/2*, and *ARID1A* were associated with worse OS in both univariable and multivariable analyses (Fig. 4E and Additional file 1: Table S12).

### Immune correlates of DNA methylation-based clusters

**Fig. 5:**
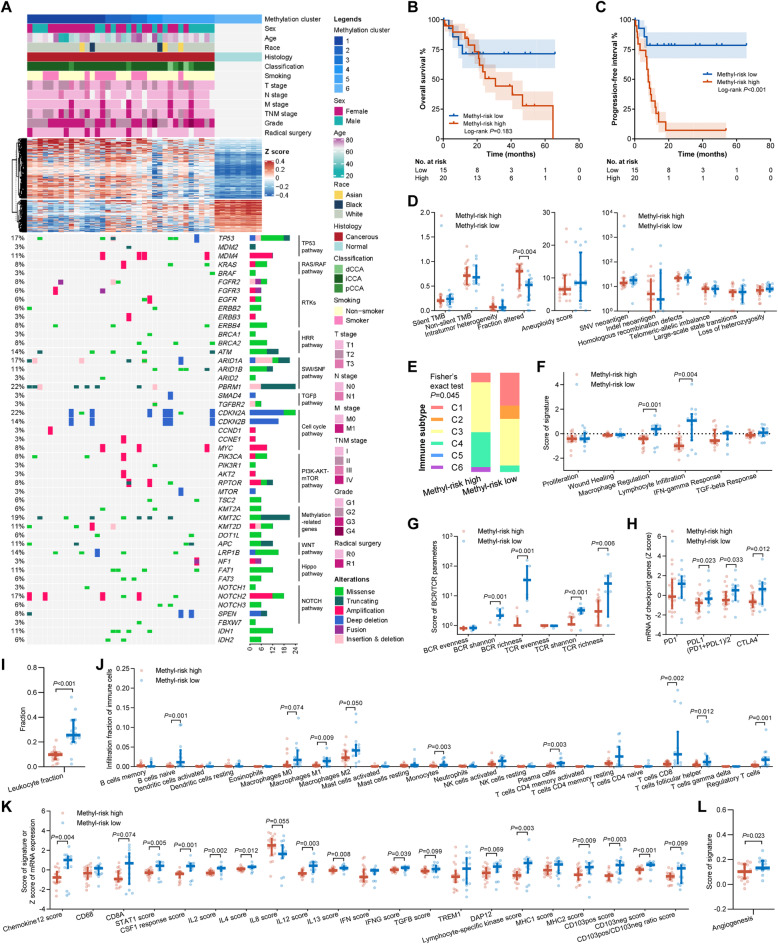
Association of global methylation changes with prognosis and immune characteristics in the TCGA-CHOL cohort. **A** Clinicopathological, methylation, and mutational data of 36 CCAs. **B**, **C** Associations of the cluster-based risk with overall survival (**B**) and progression-free interval (**C**). **D**–**L** Associations of the cluster-based risk with genomic alterations (**D**), immune subtype (**E**), the signatures for immune subtyping (**F**), BCR/TCR features (**G**), the mRNA expression of checkpoints (**H**), leukocyte fraction (**I**), the fractions of 22 immune cells (**J**), immune-related signatures (**K**), and angiogenesis signature (**L**). BCR, B cell receptor; CCA, cholangiocarcinoma; TCR, T cell receptor; TMB, tumor mutational burden; SNV, single nucleotide variation

Due to the limited publicly available datasets of GBCs with all epigenomic, transcriptomic, and survival data, we sought to firstly investigate the association between methylation and immune characteristics in a cholangiocarcinoma dataset from TCGA (TCGA-CHOL). Thirty-six cancerous tissues and nine matched normal tissues were classified into six groups via non-supervised consensus clustering (Fig. S4). Similar to the result in the EHSH cohort, gradual changes of methylation were observed ranging from cluster 1 to cluster 6 (Fig. 5A). All normal samples were in cluster 6. Clusters 1–2 and clusters 3–5 were defined as the methyl-risk high and the methyl-risk low groups, respectively. Compared to the methyl-risk high group, OS (HR = 0.47, 95% CI 0.15–1.46, *P* = 0.19, Fig. 5B) and PFI (HR = 0.14, 95% CI 0.04–0.50, *P* = 0.002, Fig. 5C) were longer in the methyl-risk low group. In multivariable analysis, consistent results were revealed (OS: multivariable HR = 0.25, 95% CI 0.05–1.31, *P* = 0.100; PFI: multivariable HR = 0.06, 95% CI 0.01–0.52, *P* = 0.011, Additional file 1: Table S13-14). Fraction altered (percentage of copy number altered chromosome regions out of measured regions) was higher in the methyl-risk high group, indicating chromatin instability (Fig. 5D).

Thorsson et al. presented immunogenomics analyses of more than 10,000 tumors in TCGA, identifying 6 immune subtypes (C1: wound healing, C2: IFN-γ dominant, C3: inflammatory, C4: lymphocyte depleted, C5: immunologically quiet, C6: TGF-β dominant) that encompass 33 cancer types based on 6 key signatures [16]. Higher frequencies of C1 and C2 and lower frequencies of C4 and C6 were observed in the methyl-risk low group (*P* = 0.045, Fig. 5E), and a lower methyl-risk was associated with higher scores of macrophage and lymphocyte signatures (Fig. 5F). As described by Thorsson et al., both C1 and C2 subtypes had low Th1/Th2 ratio, high proliferation rate, and high intratumoral heterogeneity, and C2 had the highest M1/M2 macrophage polarization, a strong CD8 signal, and the greatest TCR diversity. On the contrary, C4 displayed a more prominent macrophage signature with suppressed Th1 and high M2 response, and C6 had the highest TGF-β signature, demonstrating their immunosuppressive tumor microenvironments [16].

We further assess the association between the clusters and immune features. The samples with a lower methyl-risk exhibited higher (i) richness and Shannon index of B/T cell receptor (Fig. 5G); (ii) expression of *CD274* (PD-L1) and cytotoxic T-lymphocyte antigen 4 (CTLA-4, Fig. 5H); (iii) fraction of infiltrated leukocytes (Fig. 5I), including naïve B cell, plasma cell, monocyte, macrophage, CD8^+^ T cell, follicular helper T cell, and regulatory T cell (Fig. 5J); and (iv) multiple immune-related and angiogenesis signatures (Fig. 5K, L). Previous studies have demonstrated the pivotal role of vascular endothelial growth factor (VEGF) in interfering (i) the migration of antigen-specific T cells from the vessel into tumor and (ii) the recognition of cancer cells by cytotoxic T cells [26–28]. Generally, the angiogenesis score is negatively correlated with immune infiltration; however, we observed an overlap of greater infiltration of CD8^+^ T cell and higher angiogenesis signature in the methyl-risk low group (Fig. S5). We further calculated the signatures concerning the comparisons between naïve, effector, and exhausted CD8^+^ T cells (Fig. S6) and observed that the scores of (i) naïve vs. effector (*P* = 0.089) and (2) naïve vs. exhausted (*P* = 0.089) trended higher in the methyl-risk high subgroup, and the effector vs. exhausted score was similar in the methyl-risk high and the methyl-risk low subgroups (*P* = 0.58). These results indicate that although the samples with lower methyl-risk had more effector and exhausted CD8^+^ T cells compared to naïve CD8^+^ T cells, the quantities of effector and exhausted CD8^+^ T cells were comparable, reflecting an adaptive resistance to immune attack, which might be partially due to the higher level of angiogenesis and potentially benefit from the regimens inhibiting VEGF and immune checkpoints.

**Fig. 6:**
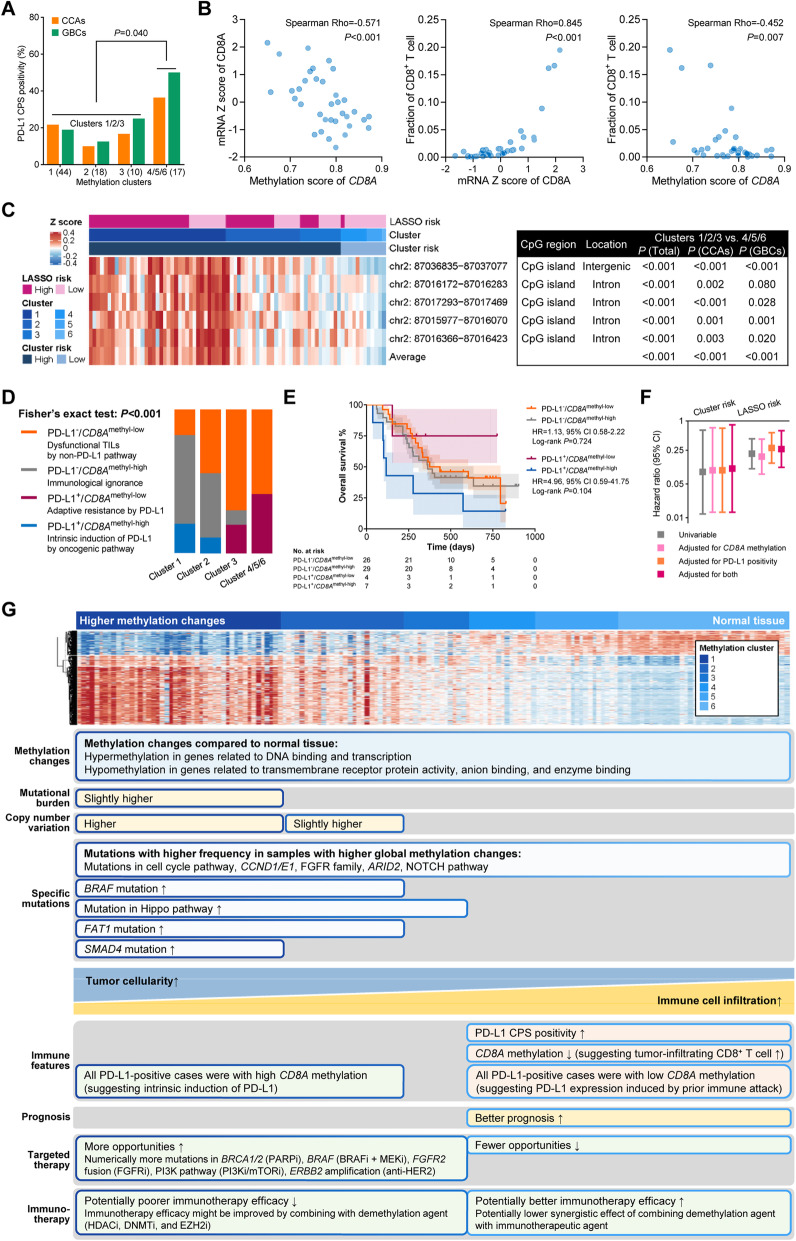
Association between global methylation changes and immune characteristics in the EHSH cohort. **A** Association between the methylation-based clusters and PD-L1 positivity (CPS score). **B** Correlations between *CD8A* methylation, CD8A mRNA, and the infiltration of CD8^+^ T cells in the TCGA-CHOL cohort. **C** Heatmap and table illustrating the methylation data and features of the five *CD8A* DMRs. **D** Associations of the methyl-risk with PD-L1 positivity and *CD8A* methylation. **E** Kaplan-Meier curves illustrating the OS of the subgroups classified by PD-L1 positivity and *CD8A* methylation. **F** Prognostic effect of the cluster-based and the LASSO-based risks after adjusting for *CD8A* methylation and/or PD-L1 positivity. **G** Representative features of the methylation-based clusters in terms of methylation changes, mutational events, immune-related characteristics, prognosis, and potential usefulness for predicting the benefits from targeted therapy and immunotherapy. CCA, cholangiocarcinoma; CPS, combined positive score; GBC, gallbladder cancer; LASSO, least absolute shrinkage and selection operator; PD-L1, programmed death-ligand 1; TMB, tumor mutational burden

Given the above findings from the TCGA-CHOL cohort, we sought to partially verify them in the EHSH cohort in terms of PD-L1 protein expression and tumor-infiltrating CD8^+^ T cells. A lower methyl-risk was associated with PD-L1 positivity (*P* = 0.040, Fig. 6A). The quantity of tumor-infiltrating CD8^+^ T cells could be reflected by *CD8A* methylation, as significant correlations were revealed between higher *CD8A* methylation, lower CD8A mRNA expression, and fewer tumor-infiltrating CD8^+^ T cells in the TCGA-CHOL cohort (Fig. 6B). In the EHSH cohort, we observed higher methylation levels of the five DMRs related to the *CD8A* gene in clusters 1/2/3 (*P* < 0.001, Fig. 6C), indicating poorer infiltration of CD8^+^ T cells in the clusters with larger methylation changes. Similar trends of the above results were shown in both the CCAs and the GBCs in the EHSH cohort, indicating the associations of methylation changes with PD-L1 expression and tumor-infiltrating CD8^+^ T cells might be a common feature in all BTCs. Given all the above immune-related results from two independent cohorts, fewer methylation changes might potentially be a novel predictor of better response to immunotherapy.

Distinguished by the density of tumor-infiltrating lymphocytes (TILs), there are two causes of PD-L1 expression: (i) prior attack by immunity (adaptive resistance) and (ii) activation of the oncogenic pathway (intrinsic induction) [29] respectively predicting better and worse benefit from immune checkpoint inhibitors (ICIs) in non-small cell lung cancer [30]. In our cohort, all PD-L1^+^ samples in clusters 1/2 had a high level of *CD8A* methylation (above median), and all PD-L1^+^ samples in clusters 3–6 had a low level of *CD8A* methylation (below median, *P* < 0.001, Fig. 6D). In addition, a large difference of OS with borderline significance was uncovered between the PD-L1^+^/*CD8A*^methyl-high^ and the PD-L1^+^/*CD8A*^methyl-low^ groups (HR = 4.96, 95% CI 0.59–41.75, *P* = 0.104, Fig. 6E), indicating the differed prognostic effects of the PD-L1 elicited by immune attack and the one induced by the oncogenic pathway. To identify the possible “PD-L1-inducing oncogenic pathway,” we revealed several enrichments of mutational event in the eleven PD-L1^+^/*CD8A*^methyl-high^ samples, including the mutations in *BRAF* (proportion = 4/11, OR = 13.1, 95% CI 2.43–71.0, *P* = 0.005), MMR pathway (proportion = 4/11, OR = 7.66, 95% CI 1.66–35.3, *P* = 0.016), Fanconi pathway (proportion = 4/11, OR = 5.31, 95% CI 1.24–22.7, *P* = 0.035), NOTCH pathway (proportion = 6/11, OR = 3.60, 95% CI 0.98–13.2, *P* = 0.053), Hippo pathway (proportion = 8/11, OR = 6.06, 95% CI 1.47–25.0, *P* = 0.010), and SWI/SNF pathway (9/11, OR = 9.59, 95% CI 1.92–48.0, *P* = 0.002). Within the SWI/SNF pathway, the mutations of BAF complex (*ARID1A*: 5/11, OR = 5.17, *P* = 0.024, and *ARID1B*: 4/11, OR = 13.1, *P* < 0.001) rather than those of PBAF complex (*ARID2*: 3/11, OR = 2.63, *P* = 0.194; *PBRM1*: 2/11, OR = 1.78, *P* = 0.395) were enriched in the PD-L1^+^/*CD8A*^methyl-high^ samples. Our results might provide novel insights into the molecular basis underlying the intrinsic induction of PD-L1 in BTCs.

Getting back to the prognostic effect of methylation changes, in order to determine whether the effect of methylation on OS is mediated by immune characteristics, we performed multivariable analysis to adjust for *CD8A* methylation and PD-L1 positivity. The association of the cluster-based risk and the LASSO-based risk with OS remained significant (Fig. 6G), indicating that the prognostic effect of DNA methylation might be partially but not completely on account of the infiltration of CD8^+^ T cells and the immune escape via PD-L1.

## Discussion

The common DNA methylation changes among BTCs may reflect their similar oncogenic mechanisms. Using these common DMRs, BTCs could be stratified into subgroups with distinct genomic aberrations, immune-related features, and survival outcomes (summarized in Fig. 6H).

Previous studies have identified the critical methylation changes during the tumorigenesis and development of CCAs and GBCs, respectively. However, no comparative study to date has identified the shared methylation features among BTCs. We identified 3369 common DMRs, and GO analysis revealed that hypomethylation mainly affected the genes related to Rho GTPase binding, transmembrane transporter activity and lyase activity, and hypermethylation targeted DNA binding and transcription. The mutations of *IDH1/2* in our EHSH cohort were rare, and none of the two mutations (*IDH1*: p. V152L and p. K4N) was at the hotspot (codon 132), which made us impossible to assess the contribution of IDH1/2 to epigenetic regulation. It is presumable that by using these common DMRs in the following analyses, we may arrive at the results generally available for all BTCs, but not limited to a specific BTC subtype at a certain anatomic location.

As for prognostication, a higher level of DNA methylation changes was independently associated with a poorer prognosis in the present study. Two previous studies mainly focusing on iCCAs reached similar results [8, 9], and we further expand its application to d/pCCAs and GBCs by using the common 3369 DMRs. Furthermore, we identified 12 significant DMRs associated with OS by LASSO regression, and the LASSO score effectively predicted OS in both training and validation sets and was identified as an independent prognostic risk factor. Compared to other prognostic risk factors, the discrimination potentials of the cluster-based risk and the LASSO-based risk were found to be superior. Of note, abundant studies have pointed out the different prognoses in GBCs, d/pCCAs, and iCCAs, which we also observed in the univariable analysis (*P* < 0.001). However, the prognostic effect of anatomic location was remarkably decreased after adjusting for TNM stage, resection margin, histological grade, CEA abnormality, and the cluster-based risk or the LASSO-based risk based on methylation changes (*P* = 0.70 and 0.98, respectively, Table 1). These results indicate that the different survivals in GBCs, d/pCCAs, and iCCAs may be largely on account of other key parameters. Moreover, the significant prognostic effects of the cluster-based risk and the LASSO-based risk in the multivariable models suggest their robustness. Combining methylation biomarkers with clinical features may further improve the prognostic estimation, which helps identify patients who would need more aggressive treatment and surveillance. However, our study was limited by sample size (*n* = 105), and further investigations to adequately assess the reliability of methylation biomarkers in clinical decision-making for BTC patients are still needed.

Of note, tumor cellularity, a confounding feature in DNA methylome studies in general, is detrimental in the study of BTCs which display desmoplasia [31]. We observed lower tumor purity in clusters 4–6 with minimal methylation changes compared to clusters 1–3 with larger methylation changes (37.5% vs. 60.0%, difference = − 22.5%) in our EHSH cohort. However, in the TCGA cohort, we also observed a higher leukocyte fraction in the clusters with minimal methylation changes to a similar extent (31.0% vs. 9.3%, difference = 21.7%). These results indicate that the difference in the tumor cellularity across different clusters may be on account of immune cell infiltration instead of desmoplasia. In addition, it is the cluster- and LASSO-based risks rather than tumor purity that were associated with OS in our analysis, and the subgroup analyses based on tumor purity and the multivariable analyses both support that the prognostic utilities of the cluster- and LASSO-based risks are not influenced by tumor purity.

In terms of precision medicine, the clusters with minimal methylation changes exhibited fewer opportunities for targeted therapy (*IDH1/2* mutation 0%, *FGFR2* fusion 0%, *BRCA1/2* mutation 6%, *ERBB2* amplification 0%, *BRAF* mutation 0%), but a hot and inflamed tumor immune microenvironment (TIME), indicating favorable benefit from immunotherapy [32–41]. More importantly, the PD-L1 expression in the methyl-risk high BTCs may be mainly induced by intrinsic activation of oncogenic pathways rather than prior immune attack. We further put forward possible candidates for this “oncogenic pathway,” including BRAF, NOTCH pathway, Hippo pathway, BAF complex, and two DNA damage repair pathways (MMR and Fanconi). On the contrary, all PD-L1-positive samples with lower methyl-risk exhibited lower *CD8A* methylation, suggesting immune attack-induced PD-L1 expression. The PD-L1^+^/*CD8A*^methyl-low^ and the PD-L1^+^/*CD8A*^methyl-high^ BTCs had different prognoses and may potentially respond to ICI treatment in different depths owing to the immune-desert properties of the PD-L1^+^/*CD8A*^methyl-high^ BTCs with no priming. In the *CD8A*^methyl-low^ BTCs, we also observed higher scores of angiogenesis signature probably suggesting stromal interactions in TIME that inhibits further immune cell infiltration, suggesting a possibility that antiangiogenic agents might further enhance the density of TILs and boost immunotherapy efficacy in the BTCs with higher CD8^+^ TILs.

Recently, Huang et al. have described the larger TIME component of Epstein-Barr virus (EBV)-associated iCCAs (EBVaICC) compared to non-EBVaICC [42]. Although the DNA methylome was not assessed in their study, plenty of evidence has pointed out the associations of EBV infection with the abnormal DNA methylation changes in solid tumors such as gastric and nasopharyngeal carcinomas [43]. Despite the rarity of EBV infection in BTCs [42], it is interesting and valuable to investigate whether the EBV-associated BTCs exhibit distinct genetic and epigenetic alterations and benefit from ICI treatment.

As for the methyl-risk high patients, immunotherapy efficacy might be improved by combining demethylation agents [44], which can induce T cell attraction and reactivation by synergistically upregulating tumor antigen presentation [45–47] and downregulating immune suppressive signals in solid tumors [48–50]. At present, all the clinical trials are in phase I/II, assessing the tolerance and efficacy of ICI plus demethylation agent [44]. More clinical and basic research probing into whether larger methylation changes can predict favorable benefits from this combination therapy is warranted.

As for limitation, first, the TNM stage was different among iCCAs, d/pCCAs, and GBCs in the present study, which may limit the exploration of the associations of anatomic location with methylation subtypes and prognosis. Given this, we performed subgroup analysis and multivariable analysis to rule out the impact from covariates including TNM stage and anatomic location on the prognostic effect of DNA methylation. Second, the sample size of the present study is not large and most samples were not obtained from advanced BTC patients, making it difficult to provide reliable explanations for the different responses to ICIs of the tumors in different anatomic locations. Third, whole-genome sequencing and gene expression data that could contribute to comprehensive molecular subtyping are missing, and this study also lacks the functional validation of specific genes in cell lines to further clarify the relation between DNA methylation and mutational events. *ARID2* mutations were enriched in clusters 1–2 with greater methylation changes, and a previous study in hepatocellular carcinoma indicates that ARID2 could recruit DNMT1 to the promoter, which increased promoter methylation [51]. Future basic studies may focus on the effect of ARID2 on the methylation in BTCs. Despite these, only based on the DNA methylation data, we successfully stratified BTC patients into subgroups with distinct prognosis and immune-related features in two independent cohorts. Importantly, consistent stratification utilities were observed in iCCAs, d/pCCAs, and GBCs, demonstrating the robustness of our findings.

## Conclusions

To our knowledge, this is the first study that identifies common DNA methylation changes of CCAs and GBCs. By leveraging the common 3369 DMRs, we subtyped the BTC patients into subgroups with distinct genomic aberrations, immune characteristics, and survival outcomes. Additionally, the 12-marker prognostic model may be used for estimating survival outcomes. Our integrative analysis based on the common DMRs provides insights into BTC pathogenesis, prognostication after curative surgery, and patient selection for immunotherapy. Conceivably, by stratification of BTCs according to molecular profiling, subtype-specific treatment modalities may be achieved in the future, which in the long term might improve the survival of BTC patients.

## Supplementary Information

**Additional file 1: Table S1**. Baseline characteristics of included 105 BTC patients. **Table S2**. Characteristics of patients with adjacent tissue. **Table S3**. Characteristics of patients with precancerous tissue. **Table S4**. List of the genes in the 520-gene panel. **Table S5**. Members of the analyzed signaling pathways. **Table S6**. Characteristics of the common differential methylated regions in BTCs. **Table S7**. Top hypermethylation regions between BTCs and adjacent/benign samples. **Table S8**. Top hypomethylation regions between BTCs and adjacent/benign samples. **Table S9**. Comparison of the clustering results using top 1,000, 500, and 250 most variable methylation DMRs. **Table S10**. Clinical correlates of methylation-based clustering in patients with survival data. **Table S11**. Detailed information of the included methylation sites in the LASSO model. **Table S12**. Association between mutational event and OS in the EHSH cohort. **Table S13**. Univariable and multivariable analyses of OS in the TCGA cohort. **Table S14**. Univariable and multivariable analyses of PFI in the TCGA cohort.

**Additional file 2: Method S1**. NGS testing for mutation. **Method S2**. Single sample gene set enrichment analysis (ssGSEA).

**Additional file 3: Fig. S1**. Consensus clustering of the EHSH cohort. Consensus matrix of non-supervised clustering of methylation signatures by K-means method (K=2-8) and delta plot assessing change in consensus cumulative distribution function area. **Fig. S2**. Feature selection using the LASSO algorithm for a prognostic model. A. The optimal tuning parameter (Lambda) in the LASSO model was selected using 3-fold cross-validation. B. LASSO coefficient profiles of the 12 features. **Fig. S3**. Association between mutational rate and TNM stage. **Fig. S4**. Consensus clustering of the TCGA-CHOL cohort. Consensus matrix of non-supervised clustering of methylation signatures by K-means method (K=2-8) and delta plot assessing change in consensus cumulative distribution function area. **Fig. S5**. Overlap of greater infiltration of CD8+ T cell and higher angiogenesis signature clustering in the TCGA-CHOL cohort. Scatter plot illustrating the infiltration of CD8 T cell and the score of angiogenesis signature in the methyl-risk high and the methyl-risk low groups. **Fig. S6**. Associations of the methyl-risk with the signatures of naïve, effector, and exhausted CD8 T cells in the TCGA-CHOL cohort. Comparisons of the signatures concerning naïve vs. effector, naïve vs. exhausted, and effector vs. exhausted between the two subgroups defined by the methyl-risk in the TCGA-CHOL cohort.

## Data Availability

The authors declare that relevant data supporting the findings of this study are available within the paper and its supplementary files. Due to ethical and privacy concerns, we are unable to publish individual-level data in our study. Raw data of DNA methylation and mutation profiling are available from the corresponding authors upon reasonable request.

## Abbreviations

AUC: Area under the curve
BTC: Biliary tract cancer
CAP: College of American Pathologist
CCA: Cholangiocarcinoma
CI: Confidence interval
CLIA: Clinical laboratory improvement amendments
CNV: Copy number variation
CTLA-4: Cytotoxic T-lymphocyte antigen 4
dCCA: Distal cholangiocarcinoma
DMR: Differentially methylated region
EHSH: Eastern hepatobiliary surgery hospital
FFPE: Formalin-fixed paraffin-embedded
GBC: Gallbladder cancer
GO: Gene Ontology
HR: Hazard ratio
iCCA: Intrahepatic cholangiocarcinoma
ICI: Immune checkpoint inhibitor
KM: Kaplan-Meier
LASSO: Least absolute shrinkage and selection operator
OS: Overall survival
pCCA: Perihilar cholangiocarcinoma
PD-L1: Programmed death-ligand 1
PFI: Progression-free interval
ROC: Receiver operating characteristic
TIL: Tumor-infiltrating lymphocyte
TIME: Tumor immune microenvironment
TMB: Tumor mutational burden

## References

[1] Lamarca A, Barriuso J, McNamara MG, Valle JW (2020). Molecular targeted therapies: ready for “prime time” in biliary tract cancer. J Hepatol.

[2] Wardell CP, Fujita M, Yamada T, Simbolo M, Fassan M, Karlic R, Polak P, Kim J, Hatanaka Y, Maejima K, Lawlor RT, Nakanishi Y, Mitsuhashi T, Fujimoto A, Furuta M, Ruzzenente A, Conci S, Oosawa A, Sasaki-Oku A, Nakano K, Tanaka H, Yamamoto Y, Michiaki K, Kawakami Y, Aikata H, Ueno M, Hayami S, Gotoh K, Ariizumi SI, Yamamoto M, Yamaue H, Chayama K, Miyano S, Getz G, Scarpa A, Hirano S, Nakamura T, Nakagawa H (2018). Genomic characterization of biliary tract cancers identifies driver genes and predisposing mutations. J Hepatol.

[3] Zou S, Li J, Zhou H, Frech C, Jiang X, Chu JS (2014). Mutational landscape of intrahepatic cholangiocarcinoma. Nat Commun.

[4] Narayan RR, Creasy JM, Goldman DA, Gonen M, Kandoth C, Kundra R (2019). Regional differences in gallbladder cancer pathogenesis: insights from a multi-institutional comparison of tumor mutations. Cancer.

[5] Nakamura H, Arai Y, Totoki Y, Shirota T, Elzawahry A, Kato M, Hama N, Hosoda F, Urushidate T, Ohashi S, Hiraoka N, Ojima H, Shimada K, Okusaka T, Kosuge T, Miyagawa S, Shibata T (2015). Genomic spectra of biliary tract cancer. Nat Genet.

[6] Lowery MA, Ptashkin R, Jordan E, Berger MF, Zehir A, Capanu M, Kemeny NE, O'Reilly EM, el-Dika I, Jarnagin WR, Harding JJ, D'Angelica MI, Cercek A, Hechtman JF, Solit DB, Schultz N, Hyman DM, Klimstra DS, Saltz LB, Abou-Alfa GK (2018). Comprehensive molecular profiling of intrahepatic and extrahepatic cholangiocarcinomas: potential targets for intervention. Clin Cancer Res.

[7] Li M, Zhang Z, Li X, Ye J, Wu X, Tan Z, Liu C, Shen B, Wang XA, Wu W, Zhou D, Zhang D, Wang T, Liu B, Qu K, Ding Q, Weng H, Ding Q, Mu J, Shu Y, Bao R, Cao Y, Chen P, Liu T, Jiang L, Hu Y, Dong P, Gu J, Lu W, Shi W, Lu J, Gong W, Tang Z, Zhang Y, Wang X, Chin YE, Weng X, Zhang H, Tang W, Zheng Y, He L, Wang H, Liu Y, Liu Y (2014). Whole-exome and targeted gene sequencing of gallbladder carcinoma identifies recurrent mutations in the ErbB pathway. Nat Genet.

[8] Jusakul A, Cutcutache I, Yong CH, Lim JQ, Huang MN, Padmanabhan N, Nellore V, Kongpetch S, Ng AWT, Ng LM, Choo SP, Myint SS, Thanan R, Nagarajan S, Lim WK, Ng CCY, Boot A, Liu M, Ong CK, Rajasegaran V, Lie S, Lim AST, Lim TH, Tan J, Loh JL, McPherson JR, Khuntikeo N, Bhudhisawasdi V, Yongvanit P, Wongkham S, Totoki Y, Nakamura H, Arai Y, Yamasaki S, Chow PKH, Chung AYF, Ooi LLPJ, Lim KH, Dima S, Duda DG, Popescu I, Broet P, Hsieh SY, Yu MC, Scarpa A, Lai J, Luo DX, Carvalho AL, Vettore AL, Rhee H, Park YN, Alexandrov LB, Gordân R, Rozen SG, Shibata T, Pairojkul C, Teh BT, Tan P (2017). Whole-genome and epigenomic landscapes of etiologically distinct subtypes of cholangiocarcinoma. Cancer Discov.

[9] Goeppert B, Toth R, Singer S, Albrecht T, Lipka DB, Lutsik P, Brocks D, Baehr M, Muecke O, Assenov Y, Gu L, Endris V, Stenzinger A, Mehrabi A, Schirmacher P, Plass C, Weichenhan D, Roessler S (2019). Integrative analysis defines distinct prognostic subgroups of intrahepatic cholangiocarcinoma. Hepatology.

[10] Farshidfar F, Zheng S, Gingras MC, Newton Y, Shih J, Robertson AG, Hinoue T, Hoadley KA, Gibb EA, Roszik J, Covington KR, Wu CC, Shinbrot E, Stransky N, Hegde A, Yang JD, Reznik E, Sadeghi S, Pedamallu CS, Ojesina AI, Hess JM, Auman JT, Rhie SK, Bowlby R, Borad MJ, Zhu AX, Stuart JM, Sander C, Akbani R, Cherniack AD, Deshpande V, Mounajjed T, Foo WC, Torbenson MS, Kleiner DE, Laird PW, Wheeler DA, McRee AJ, Bathe OF, Andersen JB, Bardeesy N, Roberts LR, Kwong LN, Akbani R, Allotey LK, Ally A, Alvaro D, Andersen JB, Appelbaum EL, Arora A, Auman JT, Balasundaram M, Balu S, Bardeesy N, Bathe OF, Baylin SB, Beroukhim R, Berrios M, Bodenheimer T, Boice L, Bootwalla MS, Borad MJ, Bowen J, Bowlby R, Bragazzi MC, Brooks D, Cardinale V, Carlsen R, Carpino G, Carvalho AL, Chaiteerakij R, Chandan VC, Cherniack AD, Chin L, Cho J, Choe G, Chuah E, Chudamani S, Cibulskis C, Cordes MG, Covington KR, Crain D, Curley E, de Rose AM, Defreitas T, Demchok JA, Deshpande V, Dhalla N, Ding L, Evason K, Farshidfar F, Felau I, Ferguson ML, Foo WC, Franchitto A, Frazer S, Fronick CC, Fulton LA, Fulton RS, Gabriel SB, Gardner J, Gastier-Foster JM, Gaudio E, Gehlenborg N, Genovese G, Gerken M, Getz G, Giama NH, Gibbs RA, Gingras MC, Giuliante F, Grazi GL, Hayes DN, Hegde AM, Heiman DI, Hess JM, Hinoue T, Hoadley KA, Holbrook A, Holt RA, Hoyle AP, Huang M, Hutter CM, Jefferys SR, Jones SJM, Jones CD, Kasaian K, Kelley RK, Kim J, Kleiner DE, Kocher JPA, Kwong LN, Lai PH, Laird PW, Lawrence MS, Leraas KM, Lichtenberg TM, Lin P, Liu W, Liu J, Lolla L, Lu Y, Ma Y, Mallery D, Mardis ER, Marra MA, Matsushita MM, Mayo M, McLellan MD, McRee AJ, Meier S, Meng S, Meyerson M, Mieczkowski PA, Miller CA, Mills GB, Moore RA, Morris S, Mose LE, Moser CD, Mounajjed T, Mungall AJ, Mungall K, Murray BA, Naresh R, Newton Y, Noble MS, O’Brien DR, Ojesina AI, Parker JS, Patel TC, Paulauskis J, Pedamallu CS, Penny R, Perou CM, Perou AH, Pihl T, Radenbaugh AJ, Ramirez NC, Rathmell WK, Reznik E, Rhie SK, Roach J, Roberts LR, Robertson AG, Sadeghi S, Saksena G, Sander C, Schein JE, Schmidt HK, Schumacher SE, Shelton C, Shelton T, Shen R, Sheth M, Shi Y, Shih J, Shinbrot E, Shroff R, Simons JV, Sipahimalani P, Skelly T, Sofia HJ, Soloway MG, Stoppler H, Stransky N, Stuart J, Sun Q, Tam A, Tan D, Tarnuzzer R, Thiessen N, Thorne LB, Torbenson MS, van den Berg DJ, Veluvolu U, Verhaak RGW, Voet D, Wan Y, Wang Z, Weinstein JN, Weisenberger DJ, Wheeler DA, Wilson RK, Wise L, Wong T, Wu CC, Wu Y, Xi L, Yang JD, Yang L, Zenklusen JC, Zhang H, Zhang J(J), Zheng S, Zmuda E (2017). Integrative genomic analysis of cholangiocarcinoma identifies distinct IDH-mutant molecular profiles. Cell Rep.

[11] O’Rourke CJ, Lafuente-Barquero J, Andersen JB (2019). Epigenome remodeling in cholangiocarcinoma. Trends Cancer.

[12] Bragelmann J, Barahona Ponce C, Marcelain K, Roessler S, Goeppert B, Gallegos I (2020). Epigenome-wide analysis of methylation changes in the sequence of gallstone disease, dysplasia, and gallbladder cancer. Hepatology.

[13] Muhammad JS, Khan MR, Ghias K (2018). DNA methylation as an epigenetic regulator of gallbladder cancer: an overview. Int J Surg.

[14] Tiwari PK (2020). Epigenetic biomarkers in gallbladder cancer. Trends Cancer.

[15] Baghel K, Kazmi HR, Chandra A, Raj S, Srivastava RN (2019). Significance of methylation status of MASPIN gene and its protein expression in prognosis of gallbladder cancer. Asia Pac J Clin Oncol.

[16] Thorsson V, Gibbs DL, Brown SD, Wolf D, Bortone DS, Ou Yang TH, Porta-Pardo E, Gao GF, Plaisier CL, Eddy JA, Ziv E, Culhane AC, Paull EO, Sivakumar IKA, Gentles AJ, Malhotra R, Farshidfar F, Colaprico A, Parker JS, Mose LE, Vo NS, Liu J, Liu Y, Rader J, Dhankani V, Reynolds SM, Bowlby R, Califano A, Cherniack AD, Anastassiou D, Bedognetti D, Mokrab Y, Newman AM, Rao A, Chen K, Krasnitz A, Hu H, Malta TM, Noushmehr H, Pedamallu CS, Bullman S, Ojesina AI, Lamb A, Zhou W, Shen H, Choueiri TK, Weinstein JN, Guinney J, Saltz J, Holt RA, Rabkin CS, Lazar AJ, Serody JS, Demicco EG, Disis ML, Vincent BG, Shmulevich I, Caesar-Johnson SJ, Demchok JA, Felau I, Kasapi M, Ferguson ML, Hutter CM, Sofia HJ, Tarnuzzer R, Wang Z, Yang L, Zenklusen JC, Zhang J(J), Chudamani S, Liu J, Lolla L, Naresh R, Pihl T, Sun Q, Wan Y, Wu Y, Cho J, DeFreitas T, Frazer S, Gehlenborg N, Getz G, Heiman DI, Kim J, Lawrence MS, Lin P, Meier S, Noble MS, Saksena G, Voet D, Zhang H, Bernard B, Chambwe N, Dhankani V, Knijnenburg T, Kramer R, Leinonen K, Liu Y, Miller M, Reynolds S, Shmulevich I, Thorsson V, Zhang W, Akbani R, Broom BM, Hegde AM, Ju Z, Kanchi RS, Korkut A, Li J, Liang H, Ling S, Liu W, Lu Y, Mills GB, Ng KS, Rao A, Ryan M, Wang J, Weinstein JN, Zhang J, Abeshouse A, Armenia J, Chakravarty D, Chatila WK, de Bruijn I, Gao J, Gross BE, Heins ZJ, Kundra R, la K, Ladanyi M, Luna A, Nissan MG, Ochoa A, Phillips SM, Reznik E, Sanchez-Vega F, Sander C, Schultz N, Sheridan R, Sumer SO, Sun Y, Taylor BS, Wang J, Zhang H, Anur P, Peto M, Spellman P, Benz C, Stuart JM, Wong CK, Yau C, Hayes DN, Parker JS, Wilkerson MD, Ally A, Balasundaram M, Bowlby R, Brooks D, Carlsen R, Chuah E, Dhalla N, Holt R, Jones SJM, Kasaian K, Lee D, Ma Y, Marra MA, Mayo M, Moore RA, Mungall AJ, Mungall K, Robertson AG, Sadeghi S, Schein JE, Sipahimalani P, Tam A, Thiessen N, Tse K, Wong T, Berger AC, Beroukhim R, Cherniack AD, Cibulskis C, Gabriel SB, Gao GF, Ha G, Meyerson M, Schumacher SE, Shih J, Kucherlapati MH, Kucherlapati RS, Baylin S, Cope L, Danilova L, Bootwalla MS, Lai PH, Maglinte DT, van den Berg DJ, Weisenberger DJ, Auman JT, Balu S, Bodenheimer T, Fan C, Hoadley KA, Hoyle AP, Jefferys SR, Jones CD, Meng S, Mieczkowski PA, Mose LE, Perou AH, Perou CM, Roach J, Shi Y, Simons JV, Skelly T, Soloway MG, Tan D, Veluvolu U, Fan H, Hinoue T, Laird PW, Shen H, Zhou W, Bellair M, Chang K, Covington K, Creighton CJ, Dinh H, Doddapaneni HV, Donehower LA, Drummond J, Gibbs RA, Glenn R, Hale W, Han Y, Hu J, Korchina V, Lee S, Lewis L, Li W, Liu X, Morgan M, Morton D, Muzny D, Santibanez J, Sheth M, Shinbrot E, Wang L, Wang M, Wheeler DA, Xi L, Zhao F, Hess J, Appelbaum EL, Bailey M, Cordes MG, Ding L, Fronick CC, Fulton LA, Fulton RS, Kandoth C, Mardis ER, McLellan MD, Miller CA, Schmidt HK, Wilson RK, Crain D, Curley E, Gardner J, Lau K, Mallery D, Morris S, Paulauskis J, Penny R, Shelton C, Shelton T, Sherman M, Thompson E, Yena P, Bowen J, Gastier-Foster JM, Gerken M, Leraas KM, Lichtenberg TM, Ramirez NC, Wise L, Zmuda E, Corcoran N, Costello T, Hovens C, Carvalho AL, de Carvalho AC, Fregnani JH, Longatto-Filho A, Reis RM, Scapulatempo-Neto C, Silveira HCS, Vidal DO, Burnette A, Eschbacher J, Hermes B, Noss A, Singh R, Anderson ML, Castro PD, Ittmann M, Huntsman D, Kohl B, le X, Thorp R, Andry C, Duffy ER, Lyadov V, Paklina O, Setdikova G, Shabunin A, Tavobilov M, McPherson C, Warnick R, Berkowitz R, Cramer D, Feltmate C, Horowitz N, Kibel A, Muto M, Raut CP, Malykh A, Barnholtz-Sloan JS, Barrett W, Devine K, Fulop J, Ostrom QT, Shimmel K, Wolinsky Y, Sloan AE, de Rose A, Giuliante F, Goodman M, Karlan BY, Hagedorn CH, Eckman J, Harr J, Myers J, Tucker K, Zach LA, Deyarmin B, Hu H, Kvecher L, Larson C, Mural RJ, Somiari S, Vicha A, Zelinka T, Bennett J, Iacocca M, Rabeno B, Swanson P, Latour M, Lacombe L, Têtu B, Bergeron A, McGraw M, Staugaitis SM, Chabot J, Hibshoosh H, Sepulveda A, Su T, Wang T, Potapova O, Voronina O, Desjardins L, Mariani O, Roman-Roman S, Sastre X, Stern MH, Cheng F, Signoretti S, Berchuck A, Bigner D, Lipp E, Marks J, McCall S, McLendon R, Secord A, Sharp A, Behera M, Brat DJ, Chen A, Delman K, Force S, Khuri F, Magliocca K, Maithel S, Olson JJ, Owonikoko T, Pickens A, Ramalingam S, Shin DM, Sica G, van Meir EG, Zhang H, Eijckenboom W, Gillis A, Korpershoek E, Looijenga L, Oosterhuis W, Stoop H, van Kessel KE, Zwarthoff EC, Calatozzolo C, Cuppini L, Cuzzubbo S, DiMeco F, Finocchiaro G, Mattei L, Perin A, Pollo B, Chen C, Houck J, Lohavanichbutr P, Hartmann A, Stoehr C, Stoehr R, Taubert H, Wach S, Wullich B, Kycler W, Murawa D, Wiznerowicz M, Chung K, Edenfield WJ, Martin J, Baudin E, Bubley G, Bueno R, de Rienzo A, Richards WG, Kalkanis S, Mikkelsen T, Noushmehr H, Scarpace L, Girard N, Aymerich M, Campo E, Giné E, Guillermo AL, van Bang N, Hanh PT, Phu BD, Tang Y, Colman H, Evason K, Dottino PR, Martignetti JA, Gabra H, Juhl H, Akeredolu T, Stepa S, Hoon D, Ahn K, Kang KJ, Beuschlein F, Breggia A, Birrer M, Bell D, Borad M, Bryce AH, Castle E, Chandan V, Cheville J, Copland JA, Farnell M, Flotte T, Giama N, Ho T, Kendrick M, Kocher JP, Kopp K, Moser C, Nagorney D, O’Brien D, O’Neill BP, Patel T, Petersen G, Que F, Rivera M, Roberts L, Smallridge R, Smyrk T, Stanton M, Thompson RH, Torbenson M, Yang JD, Zhang L, Brimo F, Ajani JA, Gonzalez AMA, Behrens C, Bondaruk J, Broaddus R, Czerniak B, Esmaeli B, Fujimoto J, Gershenwald J, Guo C, Lazar AJ, Logothetis C, Meric-Bernstam F, Moran C, Ramondetta L, Rice D, Sood A, Tamboli P, Thompson T, Troncoso P, Tsao A, Wistuba I, Carter C, Haydu L, Hersey P, Jakrot V, Kakavand H, Kefford R, Lee K, Long G, Mann G, Quinn M, Saw R, Scolyer R, Shannon K, Spillane A, Stretch, Synott M, Thompson J, Wilmott J, al-Ahmadie H, Chan TA, Ghossein R, Gopalan A, Levine DA, Reuter V, Singer S, Singh B, Tien NV, Broudy T, Mirsaidi C, Nair P, Drwiega P, Miller J, Smith J, Zaren H, Park JW, Hung NP, Kebebew E, Linehan WM, Metwalli AR, Pacak K, Pinto PA, Schiffman M, Schmidt LS, Vocke CD, Wentzensen N, Worrell R, Yang H, Moncrieff M, Goparaju C, Melamed J, Pass H, Botnariuc N, Caraman I, Cernat M, Chemencedji I, Clipca A, Doruc S, Gorincioi G, Mura S, Pirtac M, Stancul I, Tcaciuc D, Albert M, Alexopoulou I, Arnaout A, Bartlett J, Engel J, Gilbert S, Parfitt J, Sekhon H, Thomas G, Rassl DM, Rintoul RC, Bifulco C, Tamakawa R, Urba W, Hayward N, Timmers H, Antenucci A, Facciolo F, Grazi G, Marino M, Merola R, de Krijger R, Gimenez-Roqueplo AP, Piché A, Chevalier S, McKercher G, Birsoy K, Barnett G, Brewer C, Farver C, Naska T, Pennell NA, Raymond D, Schilero C, Smolenski K, Williams F, Morrison C, Borgia JA, Liptay MJ, Pool M, Seder CW, Junker K, Omberg L, Dinkin M, Manikhas G, Alvaro D, Bragazzi MC, Cardinale V, Carpino G, Gaudio E, Chesla D, Cottingham S, Dubina M, Moiseenko F, Dhanasekaran R, Becker KF, Janssen KP, Slotta-Huspenina J, Abdel-Rahman MH, Aziz D, Bell S, Cebulla CM, Davis A, Duell R, Elder JB, Hilty J, Kumar B, Lang J, Lehman NL, Mandt R, Nguyen P, Pilarski R, Rai K, Schoenfield L, Senecal K, Wakely P, Hansen P, Lechan R, Powers J, Tischler A, Grizzle WE, Sexton KC, Kastl A, Henderson J, Porten S, Waldmann J, Fassnacht M, Asa SL, Schadendorf D, Couce M, Graefen M, Huland H, Sauter G, Schlomm T, Simon R, Tennstedt P, Olabode O, Nelson M, Bathe O, Carroll PR, Chan JM, Disaia P, Glenn P, Kelley RK, Landen CN, Phillips J, Prados M, Simko J, Smith-McCune K, VandenBerg S, Roggin K, Fehrenbach A, Kendler A, Sifri S, Steele R, Jimeno A, Carey F, Forgie I, Mannelli M, Carney M, Hernandez B, Campos B, Herold-Mende C, Jungk C, Unterberg A, von Deimling A, Bossler A, Galbraith J, Jacobus L, Knudson M, Knutson T, Ma D, Milhem M, Sigmund R, Godwin AK, Madan R, Rosenthal HG, Adebamowo C, Adebamowo SN, Boussioutas A, Beer D, Giordano T, Mes-Masson AM, Saad F, Bocklage T, Landrum L, Mannel R, Moore K, Moxley K, Postier R, Walker J, Zuna R, Feldman M, Valdivieso F, Dhir R, Luketich J, Pinero EMM, Quintero-Aguilo M, Carlotti CG, Dos Santos JS, Kemp R, Sankarankuty A, Tirapelli D, Catto J, Agnew K, Swisher E, Creaney J, Robinson B, Shelley CS, Godwin EM, Kendall S, Shipman C, Bradford C, Carey T, Haddad A, Moyer J, Peterson L, Prince M, Rozek L, Wolf G, Bowman R, Fong KM, Yang I, Korst R, Rathmell WK, Fantacone-Campbell JL, Hooke JA, Kovatich AJ, Shriver CD, DiPersio J, Drake B, Govindan R, Heath S, Ley T, van Tine B, Westervelt P, Rubin MA, Lee JI, Aredes ND, Mariamidze A (2018). The immune landscape of cancer. Immunity.

[17] Mao X, Zhang Z, Zheng X, Xie F, Duan F, Jiang L, Chuai S, Han-Zhang H, Han B, Sun J (2017). Capture-based targeted ultradeep sequencing in paired tissue and plasma samples demonstrates differential subclonal ctDNA-releasing capability in advanced lung cancer. J Thorac Oncol.

[18] Wendt J, Rosenbaum H, Richmond TA, Jeddeloh JA, Burgess DL (2018). Targeted bisulfite sequencing using the SeqCap Epi Enrichment System. Methods Mol Biol.

[19] Liang N, Li B, Jia Z, Wang C, Wu P, Zheng T, Wang Y, Qiu F, Wu Y, Su J, Xu J, Xu F, Chu H, Fang S, Yang X, Wu C, Cao Z, Cao L, Bing Z, Liu H, Li L, Huang C, Qin Y, Cui Y, Han-Zhang H, Xiang J, Liu H, Guo X, Li S, Zhao H, Zhang Z (2021). Ultrasensitive detection of circulating tumour DNA via deep methylation sequencing aided by machine learning. Nat Biomed Eng.

[20] Robinson MD, Kahraman A, Law CW, Lindsay H, Nowicka M, Weber LM, Zhou X (2014). Statistical methods for detecting differentially methylated loci and regions. Front Genet.

[21] Ashburner M, Ball CA, Blake JA, Botstein D, Butler H, Cherry JM, Davis AP, Dolinski K, Dwight SS, Eppig JT, Harris MA, Hill DP, Issel-Tarver L, Kasarskis A, Lewis S, Matese JC, Richardson JE, Ringwald M, Rubin GM, Sherlock G (2000). Gene Ontology: tool for the unification of biology. The Gene Ontology Consortium. Nat Genet.

[22] Mi H, Muruganujan A, Ebert D, Huang X, Thomas PD (2019). PANTHER version 14: more genomes, a new PANTHER GO-slim and improvements in enrichment analysis tools. Nucleic Acids Res.

[23] The Gene Ontology C (2019). The Gene Ontology resource: 20 years and still GOing strong. Nucleic Acids Res.

[24] Jiao Y, Pawlik TM, Anders RA, Selaru FM, Streppel MM, Lucas DJ, Niknafs N, Guthrie VB, Maitra A, Argani P, Offerhaus GJA, Roa JC, Roberts LR, Gores GJ, Popescu I, Alexandrescu ST, Dima S, Fassan M, Simbolo M, Mafficini A, Capelli P, Lawlor RT, Ruzzenente A, Guglielmi A, Tortora G, de Braud F, Scarpa A, Jarnagin W, Klimstra D, Karchin R, Velculescu VE, Hruban RH, Vogelstein B, Kinzler KW, Papadopoulos N, Wood LD (2013). Exome sequencing identifies frequent inactivating mutations in BAP1, ARID1A and PBRM1 in intrahepatic cholangiocarcinomas. Nat Genet.

[25] Ong CK, Subimerb C, Pairojkul C, Wongkham S, Cutcutache I, Yu W, McPherson JR, Allen GE, Ng CCY, Wong BH, Myint SS, Rajasegaran V, Heng HL, Gan A, Zang ZJ, Wu Y, Wu J, Lee MH, Huang DC, Ong P, Chan-on W, Cao Y, Qian CN, Lim KH, Ooi A, Dykema K, Furge K, Kukongviriyapan V, Sripa B, Wongkham C, Yongvanit P, Futreal PA, Bhudhisawasdi V, Rozen S, Tan P, Teh BT (2012). Exome sequencing of liver fluke-associated cholangiocarcinoma. Nat Genet.

[26] Vestweber D (2015). How leukocytes cross the vascular endothelium. Nat Rev Immunol.

[27] Motz GT, Santoro SP, Wang LP, Garrabrant T, Lastra RR, Hagemann IS, Lal P, Feldman MD, Benencia F, Coukos G (2014). Tumor endothelium FasL establishes a selective immune barrier promoting tolerance in tumors. Nat Med.

[28] Jung K, Heishi T, Kowalski PS, Incio J, Rahbari NN, Khan OF (2017). Ly6Clo monocytes drive immunosuppression and confer resistance to anti-VEGFR2 cancer therapy. J Clin Invest.

[29] Sanmamed MF, Chen L (2018). A paradigm shift in cancer immunotherapy: from enhancement to normalization. Cell.

[30] Camidge DR, Doebele RC, Kerr KM (2019). Comparing and contrasting predictive biomarkers for immunotherapy and targeted therapy of NSCLC. Nat Rev Clin Oncol.

[31] Affo S, Yu LX, Schwabe RF (2017). The role of cancer-associated fibroblasts and fibrosis in liver cancer. Annu Rev Pathol.

[32] Piha-Paul SA, Oh DY, Ueno M, Malka D, Chung HC, Nagrial A, Kelley RK, Ros W, Italiano A, Nakagawa K, Rugo HS, Braud F, Varga AI, Hansen A, Wang H, Krishnan S, Norwood KG, Doi T (2020). Efficacy and safety of pembrolizumab for the treatment of advanced biliary cancer: results from the KEYNOTE-158 and KEYNOTE-028 studies. Int J Cancer.

[33] Ahn S, Lee JC, Shin DW, Kim J, Hwang JH (2020). High PD-L1 expression is associated with therapeutic response to pembrolizumab in patients with advanced biliary tract cancer. Sci Rep.

[34] Kim RD, Chung V, Alese OB, El-Rayes BF, Li D, Al-Toubah TE (2020). A phase 2 multi-institutional study of nivolumab for patients with advanced refractory biliary tract cancer. JAMA Oncol.

[35] Lin J, Yang X, Long J, Zhao S, Mao J, Wang D, Bai Y, Bian J, Zhang L, Yang X, Wang A, Xie F, Shi W, Yang H, Pan J, Hu K, Guan M, Zhao L, Huo L, Mao Y, Sang X, Wang K, Zhao H (2020). Pembrolizumab combined with lenvatinib as non-first-line therapy in patients with refractory biliary tract carcinoma. Hepatobiliary Surg Nutr.

[36] Arkenau HT, Martin-Liberal J, Calvo E, Penel N, Krebs MG, Herbst RS, Walgren RA, Widau RC, Mi G, Jin J, Ferry D, Chau I (2018). Ramucirumab plus pembrolizumab in patients with previously treated advanced or metastatic biliary tract cancer: nonrandomized, open-label, phase I trial (JVDF). Oncologist.

[37] Ueno M, Ikeda M, Morizane C, Kobayashi S, Ohno I, Kondo S, Okano N, Kimura K, Asada S, Namba Y, Okusaka T, Furuse J (2019). Nivolumab alone or in combination with cisplatin plus gemcitabine in Japanese patients with unresectable or recurrent biliary tract cancer: a non-randomised, multicentre, open-label, phase 1 study. Lancet Gastroenterol Hepatol.

[38] Loeuillard E, Yang J, Buckarma E, Wang J, Liu Y, Conboy C, Pavelko KD, Li Y, O’Brien D, Wang C, Graham RP, Smoot RL, Dong H, Ilyas S (2020). Targeting tumor-associated macrophages and granulocytic myeloid-derived suppressor cells augments PD-1 blockade in cholangiocarcinoma. J Clin Invest.

[39] Oh D-Y, Lee K-H, Lee D-W, Kim TY, Bang J-H, Nam A-R (2020). Phase II study assessing tolerability, efficacy, and biomarkers for durvalumab (D) ± tremelimumab (T) and gemcitabine/cisplatin (GemCis) in chemo-naïve advanced biliary tract cancer (aBTC). J Clin Oncol.

[40] Bader JE, Voss K, Rathmell JC (2020). Targeting metabolism to improve the tumor microenvironment for cancer immunotherapy. Mol Cell.

[41] Garris CS, Luke JJ (2020). Dendritic cells, the T-cell-inflamed tumor microenvironment, and immunotherapy treatment response. Clin Cancer Res.

[42] Huang YH, Zhang CZ, Huang QS, Yeong J, Wang F, Yang X, He YF, Zhang XL, Zhang H, Chen SL, Zheng YL, Deng R, Lin CS, Yang MM, Li Y, Jiang C, Kin-Wah Lee T, Ma S, Zeng MS, Yun JP (2021). Clinicopathologic features, tumor immune microenvironment and genomic landscape of Epstein-Barr virus-associated intrahepatic cholangiocarcinoma. J Hepatol.

[43] Cao Y, Xie L, Shi F, Tang M, Li Y, Hu J, Zhao L, Zhao L, Yu X, Luo X, Liao W, Bode AM (2021). Targeting the signaling in Epstein-Barr virus-associated diseases: mechanism, regulation, and clinical study. Signal Transduct Target Ther.

[44] Chen X, Pan X, Zhang W, Guo H, Cheng S, He Q, Yang B, Ding L (2020). Epigenetic strategies synergize with PD-L1/PD-1 targeted cancer immunotherapies to enhance antitumor responses. Acta Pharm Sin B.

[45] Wachowska M, Gabrysiak M, Muchowicz A, Bednarek W, Barankiewicz J, Rygiel T, Boon L, Mroz P, Hamblin MR, Golab J (2014). 5-Aza-2’-deoxycytidine potentiates antitumour immune response induced by photodynamic therapy. Eur J Cancer.

[46] Weber J, Salgaller M, Samid D, Johnson B, Herlyn M, Lassam N, Treisman J, Rosenberg SA (1994). Expression of the MAGE-1 tumor antigen is up-regulated by the demethylating agent 5-aza-2’-deoxycytidine. Cancer Res.

[47] Fonsatti E, Nicolay HJ, Sigalotti L, Calabro L, Pezzani L, Colizzi F (2007). Functional up-regulation of human leukocyte antigen class I antigens expression by 5-aza-2’-deoxycytidine in cutaneous melanoma: immunotherapeutic implications. Clin Cancer Res.

[48] Peng D, Kryczek I, Nagarsheth N, Zhao L, Wei S, Wang W, Sun Y, Zhao E, Vatan L, Szeliga W, Kotarski J, Tarkowski R, Dou Y, Cho K, Hensley-Alford S, Munkarah A, Liu R, Zou W (2015). Epigenetic silencing of TH1-type chemokines shapes tumour immunity and immunotherapy. Nature.

[49] Dong H, Liu S, Zhang X, Chen S, Kang L, Chen Y, Ma S, Fu X, Liu Y, Zhang H, Zou B (2019). An allosteric PRC2 inhibitor targeting EED suppresses tumor progression by modulating the immune response. Cancer Res.

[50] Nagarsheth N, Peng D, Kryczek I, Wu K, Li W, Zhao E, Zhao L, Wei S, Frankel T, Vatan L, Szeliga W, Dou Y, Owens S, Marquez V, Tao K, Huang E, Wang G, Zou W (2016). PRC2 epigenetically silences Th1-type chemokines to suppress effector T-cell trafficking in colon cancer. Cancer Res.

[51] Jiang H, Cao HJ, Ma N, Bao WD, Wang JJ, Chen TW, Zhang EB, Yuan YM, Ni QZ, Zhang FK, Ding XF, Zheng QW, Wang YK, Zhu M, Wang X, Feng J, Zhang XL, Cheng SQ, Ma DJ, Qiu L, Li JJ, Xie D (2020). Chromatin remodeling factor ARID2 suppresses hepatocellular carcinoma metastasis via DNMT1-Snail axis. Proc Natl Acad Sci U S A.

