# Supplementary material for: Common DNA methylation changes in biliary tract cancers identify subtypes with different immune characteristics and clinical outcomes
Source: BMC Med. 2022 Feb 7;20:64. doi: 10.1186/s12916-021-02197-w (PMC8822710; doi:10.1186/s12916-021-02197-w)
Supplement: Supplementary file 1 — Additional file 1: Table S1. Baseline characteristics of included 105 BTC patients. Table S2. Characteristics of patients with adjacent tissue. Table S3. Characteristics of patients with precancerous tissue. Table S4. List of the genes in the 520-gene panel. Table S5. Members of the analyzed signaling pathways. Table S6. Characteristics of the common differential methylated regions in BTCs. Table S7. Top hypermethylation regions between BTCs and adjacent/benign samples. Table S8. Top hypomethylation regions between BTCs and adjacent/benign samples. Table S9. Comparison of the clustering results using top 1,000, 500, and 250 most variable methylation DMRs. Table S10. Clinical correlates of methylation-based clustering in patients with survival data. Table S11. Detailed information of the included methylation sites in the LASSO model. Table S12. Association between mutational event and OS in the EHSH cohort. Table S13. Univariable and multivariable analyses of OS in the TCGA cohort. Table S14. Univariable and multivariable analyses of PFI in the TCGA cohort. [file 12916_2021_2197_MOESM1_ESM.docx]

**Additional file 1: Tables S1-14**

Supplement to: B. Li, Z. Qiu, Y. Xu, et al. Common DNA methylation changes of biliary tract cancer identifies subtypes with different immune characteristics and clinical outcomes.

**TABLE OF CONTENTS**

[Supplemental Tables 2](#_Toc87391461)

[Supplemental Table S1. Baseline characteristics of included 105 BTC patients. 2](#_Toc87391462)

[Supplemental Table S2. Characteristics of patients with adjacent tissue. 3](#_Toc87391463)

[Supplemental Table S3. Characteristics of patients with precancerous tissue. 4](#_Toc87391464)

[Supplemental Table S4. List of the genes in the 520-gene panel. 5](#_Toc87391465)

[Supplemental Table S5. Members of the analyzed signaling pathways. 6](#_Toc87391466)

[Supplemental Table S6. Characteristics of the common differential methylated regions in BTCs. 7](#_Toc87391467)

[Supplemental Table S7. Top hypermethylation regions between BTCs and adjacent/benign samples. 8](#_Toc87391468)

[Supplemental Table S8. Top hypomethylation regions between BTCs and adjacent/benign samples. 9](#_Toc87391469)

[Supplemental Table S9. Comparison of the clustering results using top 1,000, 500, and 250 most variable methylation DMRs. 10](#_Toc87391470)

[Supplemental Table S10. Clinical correlates of methylation-based clustering in patients with survival data. 11](#_Toc87391471)

[Supplemental Table S11. Detailed information of the included methylation sites in the LASSO model. 13](#_Toc87391472)

[Supplemental Table S12. Association between mutational event and OS in the EHSH cohort. 14](#_Toc87391473)

[Supplemental Table S13. Univariable and multivariable analyses of OS in the TCGA cohort. 15](#_Toc87391474)

[Supplemental Table S14. Univariable and multivariable analyses of PFI in the TCGA cohort. 16](#_Toc87391475)

# Supplemental Tables

## Supplemental Table S1. Baseline characteristics of included 105 BTC patients.

| Characteristic | Category | Total (n=105) | Without survival data  (n=25) | With survival data  (n=80) | P value | GBCs  (n=48) | eCCAs  (n=33) | iCCAs  (n=24) | P value |
| --- | --- | --- | --- | --- | --- | --- | --- | --- | --- |
| Age |  |  |  |  | 0.65 |  |  |  | 0.30 |
|  | <65 | 59 (56.2%) | 13 (52.0%) | 46 (57.5%) |  | 26 (54.2%) | 10 (30.3%) | 8 (33.3%) |  |
|  | ≥65 | 46 (43.8%) | 12 (48.0%) | 34 (42.5%) |  | 22 (45.8%) | 23 (69.7%) | 16 (66.7%) |  |
| Sex |  |  |  |  | 1.00 |  |  |  | 0.072 |
|  | Male | 61 (58.1%) | 15 (60.0%) | 46 (57.5%) |  | 26 (54.2%) | 22 (66.7%) | 11 (45.8%) |  |
|  | Female | 44 (41.9%) | 10 (40.0%) | 34 (42.5%) |  | 22 (45.8%) | 11 (33.3%) | 13 (54.2%) |  |
| Anatomic site |  |  |  |  | 0.17 |  |  |  |  |
|  | iCCA-small duct | 16 (15.2%) | 7 (28.0%) | 9 (11.3%) |  |  |  |  |  |
|  | iCCA-large duct | 8 (7.6%) | 2 (8.0%) | 6 (7.5%) |  |  |  |  |  |
|  | pCCA | 20 (19.0%) | 6 (24.0%) | 14 (17.5%) |  |  |  |  |  |
|  | dCCA | 13 (12.4%) | 3 (12.0%) | 10 (12.5%) |  |  |  |  |  |
|  | GBC | 48 (45.7%) | 7 (28.0%) | 41 (51.3%) |  |  |  |  |  |
| Smoking |  |  |  |  | 0.80 |  |  |  | 0.17 |
|  | Smoker | 30 (29.7%) | 8 (33.3%) | 22 (28.6%) |  | 37 (78.7%) | 19 (59.4%) | 15 (68.2%) |  |
|  | Non-smoker | 71 (70.3%) | 16 (66.7%) | 55 (71.4%) |  | 10 (21.3%) | 13 (40.6%) | 7 (31.8%) |  |
| Stage |  |  |  |  | 0.36 |  |  |  | <0.001 |
|  | I | 12 (11.7%) | 5 (20.0%) | 7 (9.0%) |  | 0 (0.0%) | 0 (0.0%) | 12 (50.0%) |  |
|  | II | 27 (26.2%) | 6 (24.0%) | 21 (26.9%) |  | 2 (4.3%) | 24 (72.7%) | 1 (4.2%) |  |
|  | III | 29 (28.2%) | 8 (32.0%) | 21 (26.9%) |  | 15 (32.6%) | 7 (21.2%) | 7 (29.2%) |  |
|  | IV | 35 (34.0%) | 6 (24.0%) | 29 (37.2%) |  | 29 (63.0%) | 2 (6.1%) | 4 (16.7%) |  |
| Radical surgery |  |  |  |  | 0.58 |  |  |  | 0.066 |
|  | R0 | 80 (78.4%) | 21 (84.0%) | 59 (76.6%) |  | 14 (31.1%) | 4 (12.1%) | 4 (16.7%) |  |
|  | R1/R2 | 22 (21.6%) | 4 (16.0%) | 18 (23.4%) |  | 31 (68.9%) | 29 (87.9%) | 20 (83.3%) |  |
| Histological grade |  |  |  |  | 0.88 |  |  |  | 0.004 |
|  | G1 | 1 (1.0%) | 0 (0.0%) | 1 (1.3%) |  | 1 (2.2%) | 0 (0.0%) | 0 (0.0%) |  |
|  | G1-G2 | 1 (1.0%) | 0 (0.0%) | 1 (1.3%) |  | 1 (2.2%) | 0 (0.0%) | 0 (0.0%) |  |
|  | G2 | 77 (74.8%) | 18 (72.0%) | 59 (75.6%) |  | 26 (56.5%) | 31 (93.9%) | 20 (83.3%) |  |
|  | G2-G3 | 11 (10.7%) | 3 (12.0%) | 8 (10.3%) |  | 8 (17.4%) | 2 (6.1%) | 1 (4.2%) |  |
|  | G3 | 13 (12.6%) | 4 (16.0%) | 9 (11.5%) |  | 10 (21.7%) | 0 (0.0%) | 3 (12.5%) |  |
| CEA (ng/mL) |  |  |  |  | 0.47 |  |  |  | 0.073 |
|  | >5 | 37 (36.3%) | 7 (29.2%) | 30 (38.5%) |  | 27 (58.7%) | 26 (78.8%) | 12 (52.2%) |  |
|  | ≤5 | 65 (63.7%) | 17 (70.8%) | 48 (61.5%) |  | 19 (41.3%) | 7 (21.2%) | 11 (47.8%) |  |
| CA19-9 (U/mL) |  |  |  |  | 0.80 |  |  |  | 0.15 |
|  | >40 | 75 (72.8%) | 19 (76.0%) | 56 (71.8%) |  | 17 (37.0%) | 6 (18.2%) | 5 (20.8%) |  |
|  | ≤40 | 28 (27.2%) | 6 (24.0%) | 22 (28.2%) |  | 29 (63.0%) | 27 (81.8%) | 19 (79.2%) |  |
| AFP (ng/mL) |  |  |  |  | 0.63 |  |  |  | 0.19 |
|  | >20 | 6 (5.7%) | 2 (8.0%) | 4 (5.0%) |  | 47 (97.9%) | 31 (93.9%) | 21 (87.5%) |  |
|  | ≤20 | 99 (94.3%) | 23 (92.0%) | 76 (95.0%) |  | 1 (2.1%) | 2 (6.1%) | 3 (12.5%) |  |
| Median follow-up (day) |  |  | 355 (236-575) |  |  |  |  |  |  |

Data are n (%) or median (IQR).

Abbreviations: AFP=alpha-fetoprotein, CA19-9=carbohydrate antigen 19-9, CEA=carcino-embryonic antigen, NA=not applicable.

## Supplemental Table S2. Characteristics of patients with adjacent tissue.

| Characteristic | Category | Adjacent bile duct  (n=22) |  | Characteristic | Category | Adjacent gallbladder  (n=28) |
| --- | --- | --- | --- | --- | --- | --- |
| Age |  |  |  | Age |  |  |
|  | <65 | 14 (63.6%) |  |  | <65 | 25 (89.3%) |
|  | ≥65 | 8 (36.4%) |  |  | ≥65 | 3 (10.7%) |
| Sex |  |  |  | Sex |  |  |
|  | Male | 11 (50.0%) |  |  | Male | 12 (42.9%) |
|  | Female | 11 (50.0%) |  |  | Female | 16 (57.1%) |
| Pathology |  |  |  | Pathology |  |  |
|  | Normal bile duct | 18 (81.8%) |  |  | Normal gallbladder | 13 (46.4%) |
|  | With cholangitis* | 4 (18.2%) |  |  | With cholecystitis | 15 (53.6%) |

Data are n (%).

*: The four adjacent bile duct tissues with cholangitis were collected from one patient with dCCA, one patient with pCCA, and two patients with iCCA.

## Supplemental Table S3. Characteristics of patients with precancerous tissue.

| Sex | Age | Anatomic site | Pathology |
| --- | --- | --- | --- |
| Male | 62 | Biliary duct | Bile duct adenoma |
| Female | 51 | Biliary duct | Bile duct adenoma |
| Female | 70 | Biliary duct | Bile duct adenoma |
| Female | 48 | Gallbladder | Gallbladder polyps |
| Male | 71 | Gallbladder | Gallbladder polyps |
| Male | 70 | Gallbladder | Gallbladder polyps |
| Female | 17 | Gallbladder | Gallbladderadenomyomatosis |
| Female | 51 | Gallbladder | Gallbladderadenomyomatosis |

## Supplemental Table S4. List of the genes in the 520-gene panel.

| **Gene list** |
| --- |
| *ABL1 ABL2 ACVR1 ACVR1B ADGRA2 AKT1 AKT2 AKT3 ALK ALOX12B ANKRD11 APC AR ARAF ARID1A ARID1B ARID2 ARID5B ASXL1 ASXL2 ATF1 ATM ATR ATRX AURKA AURKB AXIN1 AXIN2 AXL B2M BAP1 BARD1 BCL2 BCL2L1 BCL6 BCOR BCORL1 BCR BIRC3 BLM BMPR1A BRAF BRCA1 BRCA2 BRD4 BRIP1 BTK CARD11 CASP8 CBFB CBL CCND1 CCND2 CCND3 CCNE1 CD274 CD79A CD79B CDC73 CDH1 CDK12 CDK4 CDK6 CDK8 CDKN1A CDKN1B CDKN1C CDKN2A CDKN2B CDKN2C CEBPA CHD1 CHD2 CHD4 CHEK1 CHEK2 CHUK CIC CREBBP CRKL CRLF2 CSF1R CSF3R CTCF CTNNB1 CUL3 CUL4A CUL4B CYLD DAXX DDR2 DICER1 DNMT1 DNMT3A DNMT3B DOT1L EGFR EIF4E ELOC EMSY EP300 EPCAM EPHA2 EPHA3 EPHA5 EPHA7 EPHB1 ERBB2 ERBB3 ERBB4 ERCC1 ERCC2 ERG ERRFI1 ESR1 EWSR1 EZH2 FAM175A FANCA FANCC FANCD2 FANCE FANCG FANCI FANCL FAT1 FAT3 FBXW7 FCGR2B FGF12 FGF14 FGF19 FGF3 FGF4 FGFR1 FGFR2 FGFR3 FGFR4 FH FLCN FLT1 FLT3 FLT4 FOXO1 FOXP1 FRS2 FUBP1 FYN GABRA6 GALNT12 GATA1 GATA2 GATA3 GATA6 GLI1 GNA11 GNAQ GNAS GRIN2A GRM3 GSK3B GSTM1 GSTT1 H3F3A HDAC1 HDAC2 HDAC4 HGF HIST1H3F HLA-A HNF1A HNF1B HRAS HSP90AA1 IDH1 IDH2 IGF1 IGF1R IGF2 IKBKE IKZF1 IL7R INHBA INPP4A INPP4B INSR IRF2 IRF4 IRS2 JAK1 JAK2 JAK3 KAT6A KDM5A KDM5C KDM6A KDR KEAP1 KEL KIT KMT2A KMT2C KMT2D KRAS LATS1 LATS2 LMO1 LRP1B MAGI2 MAP2K1 MAP2K2 MAP2K4 MAP3K1 MAP3K13 MAPK3 MAX MCL1 MDC1 MDM2 MDM4 MED12 MEF2B MEN1 MET MITF MLH1 MLH3 MPL MRE11A MSH2 MSH3 MSH6 MST1 MST1R MTOR MUTYH MYC MYCL MYCN MYD88 NBN NCOR1 NEB NF1 NF2 NFE2L2 NFKBIA NKX2-1 NOTCH1 NOTCH2 NOTCH3 NOTCH4 NPM1 NR4A3 NRAS NRG1 NSD1 NTHL1 NTRK1 NTRK2 NTRK3 NUP93 PAK1 PAK3 PAK7 PALB2 PARK2 PARP2 PARP3 PARP4 PAX5 PBRM1 PDGFRA PDGFRB PGR PIK3C2G PIK3CA PIK3CB PIK3CD PIK3CG PIK3R1 PIK3R2 PIM1 PLCG2 PMS1 PMS2 POLD1 POLE PPP2R1A PPP2R2A PPP6C PRDM1 PREX2 PRKAR1A PRKDC PTCH1 PTEN PTK2 PTPN11 PTPRD PTPRS PTPRT QKI RAC1 RAD50 RAD51 RAD51B RAD51C RAD51D RAD52 RAD54L RAF1 RARA RASA1 RB1 RBM10 RECQL4 RET RHOA RICTOR RNF43 ROS1 RPS6KA4 RPS6KB2 RPTOR RUNX1 RUNX1T1 SDHA SDHB SDHC SDHD SETD2 SF3B1 SH2B3 SLIT2 SLX4 SMAD2 SMAD3 SMAD4 SMARCA4 SMARCB1 SMARCD1 SMO SNCAIP SOX9 SPEN SPOP SPTA1 SRC SRSF2 STAG2 STAT3 STAT5A STAT5B STK11 STK40 SUFU SYK TAF1 TBX3 TCF3 TCF7L2 TERT TET2 TGFBR2 TMPRSS2 TNFAIP3 TNFRSF14 TNFSF11 TOP1 TP53 TP63 TRAF2 TRAF7 TRRAP TSC1 TSC2 TSHR U2AF1 VEGFA VHL WRN WT1 XPO1 XRCC2 YES1 ZFHX3 ZNF217 ZNF703 ZNRF3 AMER1 APCDD1 ARFRP1 BACH1 BBC3 BCL10 BCL2L11 BCL2L2 BTG1 CALR CD276 CENPA CRBN CTLA4 CTNNA1 CXCR4 CYP17A1 DCUN1D1 DIS3 DNAJB1 E2F3 EED EGFL7 EIF1AX EIF4A2 ERCC3 ERCC4 ERCC5 FAM46C FANCF FANCM FAS FGF10 FGF23 FGF6 FGF7 FOXA1 FOXL2 GATA4 GID4 GNA13 GPS2 GREM1 H3F3B H3F3C HIST1H1C HIST1H2BD HIST1H3A HIST1H3B HIST1H3C HIST1H3D HIST1H3E HIST1H3G HIST1H3H HIST1H3I HIST1H3J HIST2H3C HIST2H3D HIST3H3 HOXB13 HSD3B1 ICOSLG ID3 IFNGR1 IL10 INHA IRS1 JUN KLF4 KLHL6 LYN LZTR1 MALT1 MAP3K14 MAPK1 MGA MYOD1 NCOA3 NEGR1 NKX3-1 PARP1 PDCD1 PDCD1LG2 PDK1 PDPK1 PHOX2B PIK3C2B PIK3C3 PIK3R3 PLK2 PMAIP1 PNRC1 POM121L12 PPM1D PRKCI PRSS8 RAB35 RAD21 RANBP2 REL RFWD2 RHEB RIT1 RPA1 RYBP SDHAF2 SH2D1A SHQ1 SOCS1 SOX10 SOX17 SOX2 STAT4 SUZ12 TACC3 TERC TET1 TGFBR1 TIPARP TMEM127 TOP2A VEGFB VEGFC VTCN1 WISP3 XIAP XRCC3 YAP1 ZBTB2 ZRSR2* |

## Supplemental Table S5. Members of the analyzed signaling pathways.

| **Pathway** | **Gene list** |
| --- | --- |
| Cell cycle | *RB1 CCNE1 CCND1 CCND2 CDK4 CDK6 CDK8 CDKN2A CDKN2B MYC CDKN1A CDKN1B CDKN1C JAK1 JAK2* |
| p53 gene | *TP53 MDM2 MDM4* |
| PI3K-AKT-mTOR pathway | *PIK3CA PIK3R1 PIK3R2 PTEN PDPK1 AKT1 AKT2 MTOR RICTOR TSC1 TSC2 RHEB RPTOR MLST8* |
| RTK signaling | *EGFR ERBB2 ERBB3 ERBB4 FGFR1 FGFR2 FGFR3 FGFR4 FGF3 FGF4 FGF12 FGF14 KIT PDGFRA PDGFRB MET VEGFA KDR* |
| Ras-Raf-MEK-Erk/JNK pathway | *KRAS HRAS NRAS BRAF RAF1 ARAF* |
| TGF-beta signaling | *TGFBR1 TGFBR2 BMPR1A ACVR1B ACVR2A SMAD2 SMAD3 SMAD4* |
| WNT signaling | *AMER1 APC CDC73 CTNNB1 GSK3B KMT2D LRP1B RNF43 SOX2 SOX9 TERT ZNRF3* |
| Hippo pathway | *FAT1 FAT3 NF1 NF2 LATS1 LATS2* |
| SWI/SNF complex | *ARID1A ARID1B ARID2 PBRM1 SMARCA1 SMARCA4 SMARCB1* |
| NOTCH signaling | *EP300 FBXW7 HDAC2 KDM5A NOTCH1 NOTCH2 NOTCH3 NOTCH4 SPEN* |
| Fanconi anemia pathway | *FANCA FANCC FANCE FANCF FANCG FANCL FANCM* |
| Homologous recombination repair | *BRCA1 BRCA2 PALB2 CHEK2 ATM RAD50 RAD52 CDK12* |
| Mismatch repair | *MLH1 PMS2 MSH2 MSH6 MSH3* |
| Nucleotide excision repair | *POLE POLD1* |
| Methylation-related genes | *DNMT3A DNMT3B DOT1L KMT2A KMT2C KMT2D TET1 TET2* |

Abbreviations: RTK=receptor tyrosine kinase, SWI/SNF=switch-sucrose nonfermentable

## Supplemental Table S6. Characteristics of the common differential methylated regions in BTCs.

|  |  | Hypermethylation (n=2534) | Hypomethylation  (n=835) | P value |
| --- | --- | --- | --- | --- |
| CpG region |  |  |  | <0.001 |
|  | CpG islands | 1931 (76.2%) | 94 (11.3%) |  |
|  | CpG shores | 377 (14.9%) | 219 (26.2%) |  |
|  | CpG shelves | 52 (2.1%) | 89 (10.7%) |  |
|  | Open sea | 174 (6.9%) | 433 (51.9%) |  |
| Location |  |  |  | <0.001 |
|  | 3-UTR exon | 42 (1.7%) | 19 (2.3%) |  |
|  | 5-UTR exon | 77 (3.0%) | 16 (1.9%) |  |
|  | CDS exon | 394 (15.5%) | 55 (6.6%) |  |
|  | Intergenic region | 726 (28.7%) | 256 (30.7%) |  |
|  | Intron | 657 (25.9%) | 309 (37.0%) |  |
|  | Non-coding exon | 80 (3.2%) | 9 (1.1%) |  |
|  | Promoter-TSS | 485 (19.1%) | 148 (17.7%) |  |
|  | TTS | 73 (2.9%) | 23 (2.8%) |  |
| Gene type of the nearest TSS |  |  |  | <0.001 |
|  | N.A. | 0 (0.0%) | 1 (0.1%) |  |
|  | ncRNA | 464 (18.3%) | 200 (24.0%) |  |
|  | Protein-coding | 1976 (78.0%) | 612 (73.3%) |  |
|  | Pseudo | 78 (3.1%) | 14 (1.7%) |  |
|  | snoRNA | 5 (0.2%) | 7 (0.8%) |  |
|  | snRNA | 11 (0.4%) | 1 (0.1%) |  |

Data are n (%).

Abbreviations: CCA=cholangiocarcinoma, CDS=coding sequence, GBC=gallbladder cancer, N.A.=not applicable, TSS=transcription start site, TTS=transcription termination site, UTR=untranslated region.

## Supplemental Table S7. Top hypermethylation regions between BTCs and adjacent/benign samples.

| No. | Mean difference  (tumor - adjacent) | Region | CpG region | Gene location | Gene of the nearest promoter |
| --- | --- | --- | --- | --- | --- |
| 1 | 0.302061 | chr4:41747657-41747974 | island:5749 | 3' UTR (NM_003924, exon 3 of 3) | *PHOX2B* |
| 2 | 0.290741 | chr8:74282708-74283007 | inter:8257 | Intergenic | *RDH10-AS1* |
| 3 | 0.284807 | chr1:146548646-146548820 | shore:2874 | intron (NM_001351365, intron 73 of 94) | *RNVU1-8* |
| 4 | 0.284079 | chr7:1094588-1095193 | shelf:14245 | promoter-TSS (NM_138445) | *GPR146* |
| 5 | 0.282622 | chr17:38607787-38607916 | inter:15379 | intron (NM_001552, intron 1 of 3) | *IGFBP4* |
| 6 | 0.282331 | chr17:50235279-50235481 | island:21099 | 5' UTR (NM_001082533, exon 2 of 10) | *CA10* |
| 7 | 0.279145 | chr8:101821864-101822132 | inter:8366 | Intergenic | *PABPC1* |
| 8 | 0.277707 | chr1:243646151-243646800 | island:2406 | intron (NM_001350248, intron 17 of 18) | *MIR4677* |
| 9 | 0.276343 | chr1:91182731-91183127 | island:1309 | promoter-TSS (NM_020063) | *BARHL2* |
| 10 | 0.275671 | chr2:200327260-200327423 | island:3719 | intron (NM_015265, intron 1 of 11) | *SATB2* |
| 11 | 0.274901 | chr15:70458192-70458291 | inter:13701 | Intergenic | *TLE3* |
| 12 | 0.274487 | chr6:26225268-26225776 | island:7778 | exon (NM_003532, exon 1 of 1) | *HIST1H3E* |
| 13 | 0.274347 | chr19:58715263-58716108 | island:24721 | intron (NM_001278734, intron 4 of 8) | *ZNF274* |
| 14 | 0.273161 | chr5:172175595-172175765 | island:7364 | Intergenic | *LOC101928093* |
| 15 | 0.27296 | chr7:4832039-4832602 | island:9099 | 3' UTR (NM_014855, exon 17 of 17) | *MIR4656* |
| 16 | 0.271252 | chr6:10395818-10398916 | island:7673 | 3' UTR (NM_003220, exon 7 of 7) | *TFAP2A-AS2* |
| 17 | 0.270979 | chr17:46799384-46800038 | shore:38332 | promoter-TSS (NM_001282275) | *PRAC1* |
| 18 | 0.270507 | chr2:20870758-20871212 | island:2632 | exon (NM_182828, exon 2 of 2) | *GDF7* |
| 19 | 0.269681 | chr8:65498582-65498650 | island:10898 | TTS (NM_001324112) | *LOC401463* |
| 20 | 0.267971 | chr20:33585040-33585243 | island:25050 | exon (NM_020884, exon 32 of 45) | *MIR499B* |
| 21 | 0.267828 | chr17:46803877-46804528 | island:21020 | exon (NM_006361, exon 2 of 2) | *HOXB13* |
| 22 | 0.266768 | chr2:63282378-63283434 | island:2890 | non-coding (NR_130153, exon 5 of 5) | *OTX1* |
| 23 | 0.265738 | chr15:53084654-53087699 | island:18101 | Intergenic | *ONECUT1* |
| 24 | 0.265613 | chr8:145925455-145925533 | island:11427 | Intergenic | *ARHGAP39* |
| 25 | 0.2644 | chr15:53087831-53087988 | shore:33123 | Intergenic | *ONECUT1* |
| 26 | 0.263267 | chr20:37352983-37353286 | island:25113 | promoter-TSS (NM_080552) | *SLC32A1* |
| 27 | 0.262884 | chr2:105472193-105472456 | island:3195 | exon (NM_006236, exon 1 of 1) | *POU3F3* |
| 28 | 0.262208 | chr1:146549240-146551009 | island:1563 | intron (NM_001351365, intron 73 of 94) | *RNVU1-8* |
| 29 | 0.261232 | chr1:165324131-165324216 | island:1885 | intron (NM_177398, intron 2 of 8) | *LMX1A* |
| 30 | 0.259455 | chr10:45923322-45923400 | island:12978 | intron (NM_000698, intron 6 of 13) | *LOC102724323* |
| 31 | 0.258887 | chr2:68545929-68546139 | shore:5394 | intron (NM_001111101, intron 1 of 2) | *CNRIP1* |
| 32 | 0.257373 | chr12:64215500-64215989 | island:15752 | non-coding (NR_126167, exon 1 of 4) | *RXYLT1-AS1* |
| 33 | 0.257336 | chr20:34190087-34190146 | island:25063 | non-coding (NR_119376, exon 12 of 44) | *FER1L4* |
| 34 | 0.255983 | chr11:109292789-109292982 | shore:27309 | promoter-TSS (NM_207645) | *C11orf87* |
| 35 | 0.255691 | chr20:2780763-2780856 | shore:44873 | intron (NM_001184699, intron 1 of 13) | *CPXM1* |
| 36 | 0.253889 | chr19:19625158-19625504 | island:23519 | 3' UTR (NM_032037, exon 1 of 1) | *TSSK6* |
| 37 | 0.253789 | chr1:92945805-92947173 | island:1329 | exon (NM_001127215, exon 4 of 7) | *GFI1* |
| 38 | 0.253151 | chr2:91634973-91635222 | island:3090 | Intergenic | *LOC654342* |
| 39 | 0.252912 | chr1:237205272-237206115 | island:2384 | promoter-TSS (NM_001035) | *RYR2* |
| 40 | 0.252783 | chr12:2862340-2862392 | island:15213 | intron (NR_146317, intron 3 of 3) | *FKBP4* |
| 41 | 0.252708 | chr8:72468818-72468881 | island:10930 | Intergenic | *EYA1* |
| 42 | 0.251119 | chr7:73786697-73787395 | shelf:15298 | intron (NM_032421, intron 8 of 15) | *GTF2IRD1* |
| 43 | 0.250633 | chr1:152080228-152081620 | island:1695 | exon (NM_007113, exon 3 of 3) | *TCHH* |
| 44 | 0.250281 | chr17:6617124-6617227 | island:20260 | promoter-TSS (NM_001284509) | *SLC13A5* |
| 45 | 0.250208 | chr4:134071663-134071772 | island:6102 | exon (NM_020815, exon 1 of 1) | *PCDH10* |
| 46 | 0.250132 | chr7:6703571-6704236 | island:9190 | Intergenic | *ZNF316* |
| 47 | 0.249655 | chr12:53465904-53466024 | inter:11717 | intron (NM_032840, intron 6 of 10) | *SPRYD3* |
| 48 | 0.249636 | chr12:5019337-5019401 | island:15238 | 5' UTR (NM_000217, exon 1 of 2) | *KCNA1* |
| 49 | 0.249328 | chr14:60973976-60974242 | island:17287 | Intergenic | *SIX6* |
| 50 | 0.248842 | chr14:38080153-38080514 | island:17175 | intron (NM_001310135, intron 2 of 32) | *TTC6* |

## Supplemental Table S8. Top hypomethylation regions between BTCs and adjacent/benign samples.

| No. | Mean difference  (tumor - adjacent) | Region | CpG region | Gene location | Gene of the nearest promoter |
| --- | --- | --- | --- | --- | --- |
| 1 | -0.30201 | chr3:115510372-115510898 | inter:3704 | Intergenic | *SNORD155* |
| 2 | -0.2982 | chr2:234881339-234881681 | inter:3062 | intron (NM_024080, intron 17 of 25) | *TRPM8* |
| 3 | -0.29415 | chr14:105103494-105103918 | island:17690 | Intergenic | *LINC02280* |
| 4 | -0.29329 | chr1:244964768-244964829 | inter:1837 | Intergenic | *COX20* |
| 5 | -0.28959 | chr4:2432194-2432327 | island:5490 | intron (NM_001193282, intron 2 of 15) | *CFAP99* |
| 6 | -0.28927 | chr17:76183632-76184081 | shore:39036 | promoter-TSS (NM_001346663) | *AFMID* |
| 7 | -0.2887 | chr19:49541882-49542215 | inter:17458 | Intergenic | *SNAR-G1* |
| 8 | -0.28594 | chr4:7755069-7755425 | inter:4313 | promoter-TSS (NR_026892) | *AFAP1-AS1* |
| 9 | -0.28371 | chr22:41048467-41048581 | inter:18884 | Intergenic | *MKL1* |
| 10 | -0.28304 | chr12:124718338-124718481 | inter:12157 | intron (NM_001347902, intron 3 of 4) | *RFLNA* |
| 11 | -0.28021 | chr1:161283493-161283673 | shore:3408 | promoter-TSS (NM_003001) | *SDHC* |
| 12 | -0.28016 | chr1:179334176-179334333 | shore:3598 | promoter-TSS (NR_073544) | *AXDND1* |
| 13 | -0.27826 | chr8:142984528-142984725 | island:11222 | Intergenic | *MIR4539* |
| 14 | -0.27702 | chr12:121714786-121715044 | shelf:25472 | intron (NM_153499, intron 1 of 15) | *CAMKK2* |
| 15 | -0.27678 | chr1:151486421-151486560 | shelf:2635 | intron (NM_020770, intron 1 of 20) | *CGN* |
| 16 | -0.27677 | chr8:128306812-128306986 | inter:8445 | intron (NR_117100, intron 5 of 5) | *CASC21* |
| 17 | -0.27606 | chr1:155534485-155534683 | shore:3238 | TTS (NR_147963) | *ASH1L* |
| 18 | -0.27466 | chr6:74160206-74160337 | shore:15173 | intron (NM_138441, intron 1 of 4) | *CGAS* |
| 19 | -0.27294 | chr11:67745857-67746013 | inter:10950 | Intergenic | *UNC93B1* |
| 20 | -0.27106 | chr1:220961001-220961102 | shore:4067 | intron (NM_022746, intron 1 of 6) | *MARCH1* |
| 21 | -0.2706 | chr7:80549660-80550531 | shore:17808 | intron (NM_001350120, intron 1 of 17) | *SEMA3C* |
| 22 | -0.2702 | chr12:30947711-30947918 | shore:28166 | promoter-TSS (NR_040245) | *LINC00941* |
| 23 | -0.26683 | chr1:201366374-201366459 | shelf:3239 | intron (NM_005558, intron 1 of 9) | *LAD1* |
| 24 | -0.26661 | chr17:25878832-25879003 | inter:15195 | intron (NM_014238, intron 3 of 20) | *LGALS9* |
| 25 | -0.26634 | chr11:71326510-71326913 | inter:11006 | Intergenic | *KRTAP5-11* |
| 26 | -0.26634 | chr17:43448448-43448924 | shore:38177 | Intergenic | *MAP3K14* |
| 27 | -0.26564 | chr20:62111136-62111186 | shore:46073 | Intergenic | *KCNQ2* |
| 28 | -0.26539 | chr9:140400741-140400891 | shore:23109 | intron (NM_001098537, intron 12 of 34) | *PNPLA7* |
| 29 | -0.26527 | chr17:39780974-39781230 | inter:15385 | promoter-TSS (NM_000422) | *KRT17* |
| 30 | -0.26521 | chr7:39760370-39760422 | inter:7062 | Intergenic | *LINC00265* |
| 31 | -0.26444 | chr13:114770495-114770825 | shelf:26752 | intron (NM_001320822, intron 18 of 23) | *RASA3* |
| 32 | -0.26354 | chr6:1267492-1267647 | inter:5795 | Intergenic | *FOXQ1* |
| 33 | -0.26343 | chr10:135123006-135123108 | island:13779 | promoter-TSS (NM_001256617) | *TUBGCP2* |
| 34 | -0.26305 | chr13:114880671-114880726 | shore:31061 | intron (NM_001320821, intron 1 of 25) | *RASA3* |
| 35 | -0.26198 | chr8:102039452-102039679 | inter:8367 | Intergenic | *SNORD155* |
| 36 | -0.26182 | chr3:195869929-195870288 | inter:4130 | intron (NR_034088, intron 1 of 2) | *TRPM8* |
| 37 | -0.26152 | chr2:10054851-10055019 | inter:1958 | intron (NM_001318976, intron 12 of 14) | *LINC02280* |
| 38 | -0.26152 | chr17:79694665-79694771 | inter:15935 | Intergenic | *COX20* |
| 39 | -0.26146 | chr16:2234671-2235174 | shore:34459 | intron (NM_020764, intron 13 of 19) | *CFAP99* |
| 40 | -0.26045 | chr7:97580714-97580823 | inter:7394 | intron (NR_147989, intron 1 of 18) | *AFMID* |
| 41 | -0.26043 | chr8:144254725-144255026 | inter:8530 | Intergenic | *SNAR-G1* |
| 42 | -0.26007 | chr15:81134721-81134843 | inter:13810 | promoter-TSS (NR_030393) | *AFAP1-AS1* |
| 43 | -0.25994 | chr7:11012871-11012994 | shore:16859 | promoter-TSS (NR_033436) | *MKL1* |
| 44 | -0.25982 | chr7:5647961-5648260 | island:9152 | Intergenic | *RFLNA* |
| 45 | -0.25903 | chr8:98862120-98862193 | inter:8347 | intron (NM_018407, intron 6 of 6) | *SDHC* |
| 46 | -0.25889 | chr7:54899506-54899729 | shore:17352 | Intergenic | *AXDND1* |
| 47 | -0.25735 | chr10:98129682-98130386 | inter:10052 | exon (NM_012465, exon 20 of 21) | *MIR4539* |
| 48 | -0.2572 | chr5:2854134-2854201 | inter:4986 | Intergenic | *CAMKK2* |
| 49 | -0.25639 | chr2:127955156-127955380 | inter:2590 | intron (NM_001001665, intron 4 of 7) | *CGN* |
| 50 | -0.25639 | chr7:1968172-1968380 | shore:16527 | intron (NM_001013836, intron 17 of 18) | *CASC21* |

## Supplemental Table S9. Comparison of the clustering results using top 1,000, 500, and 250 most variable methylation DMRs.

|  | **Top 1,000** | **Top 500** | **Top 250** | **No.** |
| --- | --- | --- | --- | --- |
| 1 | Methyl-risk high | Methyl-risk high | Methyl-risk high | 84 |
| 2 | Methyl-risk high | Methyl-risk high | Methyl-risk low | 1 |
| 3 | Methyl-risk high | Methyl-risk low | Methyl-risk high | 2 |
| 4 | Methyl-risk high | Methyl-risk low | Methyl-risk low | 3 |
| 5 | Methyl-risk low | Methyl-risk high | Methyl-risk high | 1 |
| 6 | Methyl-risk low | Methyl-risk high | Methyl-risk low | 0 |
| 7 | Methyl-risk low | Methyl-risk low | Methyl-risk high | 0 |
| 8 | Methyl-risk low | Methyl-risk low | Methyl-risk low | 72 |
| Total |  |  |  | 163 |

## Supplemental Table S10. Clinical correlates of methylation-based clustering in patients with survival data.

|  |  | Cluster 1 | Cluster 2 | Cluster 3 | Cluster 4/5/6 | P value |
| --- | --- | --- | --- | --- | --- | --- |
| Sex |  |  |  |  |  | 0.32 |
|  | Male | 26 (56.5%) | 16 (61.5%) | 10 (76.9%) | 9 (45.0%) |  |
|  | Female | 20 (43.5%) | 10 (38.5%) | 3 (23.1%) | 11 (55.0%) |  |
| Age |  |  |  |  |  | 0.59 |
|  | <65 | 23 (50.0%) | 16 (61.5%) | 9 (69.2%) | 11 (55.0%) |  |
|  | ≥65 | 23 (50.0%) | 10 (38.5%) | 4 (30.8%) | 9 (45.0%) |  |
| Smoking |  |  |  |  |  | 0.59 |
|  | Non-smoker | 30 (66.7%) | 21 (80.8%) | 7 (63.6%) | 13 (68.4%) |  |
|  | Smoker | 15 (33.3%) | 5 (19.2%) | 4 (36.4%) | 6 (31.6%) |  |
| Anatomic site |  |  |  |  |  | 0.72 |
|  | iCCA-small duct | 7 (15.2%) | 6 (23.1%) | 2 (15.4%) | 1 (5.0%) |  |
|  | iCCA-large duct | 2 (4.3%) | 2 (7.7%) | 2 (15.4%) | 2 (10.0%) |  |
|  | pCCA | 9 (19.6%) | 4 (15.4%) | 1 (7.7%) | 6 (30.0%) |  |
|  | dCCA | 5 (10.9%) | 3 (11.5%) | 1 (7.7%) | 4 (20.0%) |  |
|  | Gallbladder cancer | 23 (50.0%) | 11 (42.3%) | 7 (53.8%) | 7 (35.0%) |  |
| TNM stage |  |  |  |  |  | 0.38 |
|  | I | 3 (6.8%) | 5 (19.2%) | 3 (23.1%) | 1 (5.0%) |  |
|  | II | 10 (22.7%) | 7 (26.9%) | 2 (15.4%) | 8 (40.0%) |  |
|  | III | 12 (27.3%) | 6 (23.1%) | 4 (30.8%) | 7 (35.0%) |  |
|  | IV | 19 (43.2%) | 8 (30.8%) | 4 (30.8%) | 4 (20.0%) |  |
| Histological grade |  |  |  |  |  | 0.29 |
|  | G1 | 1 (2.2%) | 0 (0.0%) | 0 (0.0%) | 0 (0.0%) |  |
|  | G1-G2 | 1 (2.2%) | 0 (0.0%) | 0 (0.0%) | 0 (0.0%) |  |
|  | G2 | 35 (76.1%) | 16 (61.5%) | 8 (61.5%) | 18 (90.0%) |  |
|  | G2-G3 | 5 (10.9%) | 3 (11.5%) | 3 (23.1%) | 0 (0.0%) |  |
|  | G3 | 3 (6.5%) | 7 (26.9%) | 1 (7.7%) | 2 (10.0%) |  |
|  | Gx | 1 (2.2%) | 0 (0.0%) | 1 (7.7%) | 0 (0.0%) |  |
| Radical surgery |  |  |  |  |  | 0.76 |
|  | R0 | 35 (77.8%) | 19 (79.2%) | 9 (69.2%) | 17 (85.0%) |  |
|  | R1/R2 | 10 (22.2%) | 5 (20.8%) | 4 (30.8%) | 3 (15.0%) |  |
| CEA (ng/mL) |  |  |  |  |  | 0.007 |
|  | ≤5 | 25 (56.8%) | 18 (69.2%) | 4 (33.3%) | 18 (90.0%) |  |
|  | >5 | 19 (43.2%) | 8 (30.8%) | 8 (66.7%) | 2 (10.0%) |  |
| CA19-9 (U/mL) |  |  |  |  |  | 0.44 |
|  | ≤40 | 10 (22.7%) | 10 (38.5%) | 4 (30.8%) | 4 (20.0%) |  |
|  | >40 | 34 (77.3%) | 16 (61.5%) | 9 (69.2%) | 16 (80.0%) |  |
| AFP (ng/mL) |  |  |  |  |  | 0.53 |
|  | ≤20 | 42 (91.3%) | 25 (96.2%) | 12 (92.3%) | 20 (100.0%) |  |
|  | >20 | 4 (8.7%) | 1 (3.8%) | 1 (7.7%) | 0 (0.0%) |  |
| Hep-1 |  |  |  |  |  | 0.65 |
|  | Negative | 33 (91.7%) | 22 (95.7%) | 10 (90.9%) | 15 (100.0%) |  |
|  | Positive | 3 (8.3%) | 1 (4.3%) | 1 (9.1%) | 0 (0.0%) |  |
| MUC-1 |  |  |  |  |  | 0.65 |
|  | Negative | 33 (91.7%) | 22 (95.7%) | 10 (90.9%) | 15 (100.0%) |  |
|  | Positive | 3 (8.3%) | 1 (4.3%) | 1 (9.1%) | 0 (0.0%) |  |
| CK19 |  |  |  |  |  | 0.069 |
|  | Negative | 1 (2.2%) | 4 (16.0%) | 1 (8.3%) | 0 (0.0%) |  |
|  | Positive | 45 (97.8%) | 21 (84.0%) | 11 (91.7%) | 19 (100.0%) |  |
| HER2 |  |  |  |  |  | 0.67 |
|  | Negative | 14 (77.8%) | 9 (100.0%) | 3 (60.0%) | 2 (66.7%) |  |
|  | Positive | 4 (22.2%) | 0 (0.0%) | 2 (40.0%) | 1 (33.3%) |  |
| VI |  |  |  |  |  | 0.29 |
|  | Negative | 22 (100.0%) | 8 (88.9%) | 5 (100.0%) | 6 (100.0%) |  |
|  | Positive | 0 (0.0%) | 1 (11.1%) | 0 (0.0%) | 0 (0.0%) |  |
| P63 |  |  |  |  |  | 0.11 |
|  | Negative | 19 (82.6%) | 7 (63.6%) | 7 (100.0%) | 3 (50.0%) |  |
|  | Positive | 4 (17.4%) | 4 (36.4%) | 0 (0.0%) | 3 (50.0%) |  |
| GPC-3 |  |  |  |  |  | 0.49 |
|  | Negative | 41 (97.6%) | 20 (95.2%) | 8 (88.9%) | 16 (100.0%) |  |
|  | Positive | 1 (2.4%) | 1 (4.8%) | 1 (11.1%) | 0 (0.0%) |  |
| CgA |  |  |  |  |  | 0.83 |
|  | Negative | 22 (95.7%) | 9 (100.0%) | 5 (100.0%) | 6 (100.0%) |  |
|  | Positive | 1 (4.3%) | 0 (0.0%) | 0 (0.0%) | 0 (0.0%) |  |
| CK5/6 |  |  |  |  |  | 0.61 |
|  | Negative | 23 (59.0%) | 12 (66.7%) | 7 (77.8%) | 9 (52.9%) |  |
|  | Positive | 16 (41.0%) | 6 (33.3%) | 2 (22.2%) | 8 (47.1%) |  |
| S100 |  |  |  |  |  | N.A. |
|  | Negative | 22 (100.0%) | 11 (100.0%) | 5 (100.0%) | 6 (100.0%) |  |
|  | Positive | 0 (0.0%) | 0 (0.0%) | 0 (0.0%) | 0 (0.0%) |  |
| CAM5.2 |  |  |  |  |  | 0.063 |
|  | Negative | 0 (0.0%) | 2 (11.1%) | 0 (0.0%) | 0 (0.0%) |  |
|  | Positive | 39 (100.0%) | 16 (88.9%) | 8 (100.0%) | 17 (100.0%) |  |
| EMA |  |  |  |  |  | 0.065 |
|  | Negative | 1 (4.2%) | 3 (30.0%) | 0 (0.0%) | 0 (0.0%) |  |
|  | Positive | 23 (95.8%) | 7 (70.0%) | 5 (100.0%) | 6 (100.0%) |  |

Data are n (%).

Abbreviations: AFP=alpha-fetoprotein, CA19-9=carbohydrate antigen 19-9, CCA=cholangiocarcinoma, CEA=carcino-embryonic antigen.

## Supplemental Table S11. Detailed information of the included methylation sites in the LASSO model.

|  | Coefficient | CpG region | Annotation | Gene | Gene type |
| --- | --- | --- | --- | --- | --- |
| chr13: 28496082-28497539 | 0.3149 | CpG islands | intron (NM_000209, intron 1 of 1) | *PDX1* | protein-coding |
| chr8: 142157078-142157173 | 1.7128 | Open sea | intron (NR_148197, intron 5 of 23) | *DENND3* | protein-coding |
| chr19: 39906359-39906454 | 2.2218 | CpG shores | intron (NM_001351693, intron 4 of 19) | *PLEKHG2* | protein-coding |
| chr6: 42695707-42695779 | 1.553 | CpG shores | non-coding (NR_037141, exon 1 of 1) | *ATP6V0CP3* | pseudo |
| chr12: 31079986-31080065 | 0.964 | CpG shores | intron (NM_001080509, intron 1 of 7) | *TSPAN11* | protein-coding |
| chr15: 70458192-70458291 | 0.9083 | Open sea | Intergenic | *TLE3* | protein-coding |
| chr12: 63193951-63194049 | -0.7286 | CpG shores | intron (NM_020700, intron 3 of 9) | *PPM1H* | protein-coding |
| chr1: 151509750-151509825 | -0.9558 | CpG shelves | exon (NM_020770, exon 21 of 21) | *TUFT1* | protein-coding |
| chr2: 242491782-242491851 | -0.6939 | Open sea | intron (NR_033346, intron 1 of 1) | *BOK* | protein-coding |
| chr19: 4556944-4556998 | -0.2026 | CpG shores | exon (NM_032108, exon 5 of 17) | *SEMA6B* | protein-coding |
| chr16: 86653176-86653260 | -0.8409 | Open sea | Intergenic | *FOXL1* | protein-coding |
| chr13: 61047960-61048030 | -1.0514 | Open sea | intron (NM_001146070, intron 5 of 13) | *TDRD3* | protein-coding |

## Supplemental Table S12. Association between mutational event and OS in the EHSH cohort.

| **Mutation** | **Univariable P** | **95% CI** | **Multivariable P** | **95% CI** |
| --- | --- | --- | --- | --- |
| CNV>2 | 0.842 | 0.94 (0.49-1.80) | 0.225 | 0.62 (0.29-1.34) |
| **TP53 pathway** | 0.742 | 1.12 (0.56-2.24) | 0.656 | 0.84 (0.38-1.83) |
| *TP53* | 0.953 | 0.98 (0.53-1.82) | 0.258 | 0.66 (0.32-1.35) |
| **RAS pathway** | 0.509 | 0.80 (0.41-1.56) | 0.563 | 1.27 (0.57-2.81) |
| *KRAS* | 0.262 | 0.67 (0.33-1.36) | 0.521 | 1.31 (0.58-2.98) |
| *BRAF* | 0.001 | 4.82 (1.83-12.72) | <0.001 | 14.17 (4.33-46.37) |
| **Cell cycle pathway** | 0.910 | 1.04 (0.56-1.91) | 0.477 | 0.77 (0.37-1.60) |
| *CDKN2A/B* | 0.147 | 0.42 (0.13-1.36) | 0.483 | 0.58 (0.13-2.64) |
| *CCND1/E1* | 0.320 | 1.44 (0.70-2.93) | 0.535 | 0.77 (0.34-1.74) |
| **RTKs** | 0.174 | 0.65 (0.35-1.21) | 0.337 | 0.70 (0.34-1.44) |
| ERBB family | 0.501 | 0.77 (0.37-1.63) | 0.722 | 0.86 (0.39-1.94) |
| *ERBB2* | 0.745 | 0.82 (0.25-2.67) | 0.187 | 0.37 (0.09-1.62) |
| FGFR family | 0.887 | 0.95 (0.44-2.05) | 0.558 | 0.77 (0.33-1.83) |
| **HRR pathway** | 0.983 | 0.99 (0.51-1.94) | 0.252 | 0.63 (0.28-1.39) |
| *BRCA1/2* | 0.866 | 0.91 (0.33-2.57) | 0.072 | 0.34 (0.11-1.10) |
| *ATM* | 0.504 | 1.32 (0.59-2.97) | 0.754 | 1.16 (0.46-2.91) |
| **MMR pathway** | 0.012 | 3.83 (1.35-10.87) | 0.024 | 4.99 (1.24-20.12) |
| **NER pathway** | 0.198 | 1.97 (0.70-5.56) | 0.340 | 2.15 (0.45-10.34) |
| **Fanconi pathway** | 0.871 | 1.10 (0.34-3.57) | 0.546 | 1.46 (0.43-5.04) |
| **PI3K pathway** | 0.848 | 0.94 (0.48-1.83) | 0.810 | 0.91 (0.41-1.99) |
| **TGFβ pathway** | 0.741 | 0.89 (0.46-1.75) | 0.818 | 1.09 (0.51-2.36) |
| *SMAD4* | 0.119 | 0.50 (0.21-1.20) | 0.172 | 0.51 (0.19-1.34) |
| *TGFBR1/2* | 0.074 | 2.59 (0.91-7.36) | 0.003 | 5.58 (1.77-17.58) |
| **SWI/SNF complex** | 0.109 | 1.66 (0.89-3.09) | 0.157 | 1.74 (0.81-3.76) |
| **BAF complex** | 0.256 | 1.54 (0.73-3.23) | 0.392 | 1.50 (0.59-3.79) |
| *ARID1A* | 0.037 | 2.41 (1.06-5.49) | 0.001 | 5.70 (1.97-16.55) |
| *ARID1B* | 0.524 | 0.63 (0.15-2.63) | 0.139 | 0.30 (0.06-1.48) |
| **PBAF complex** | 0.185 | 1.61 (0.80-3.24) | 0.313 | 1.55 (0.66-3.62) |
| *ARID2* | 0.390 | 1.43 (0.63-3.27) | 0.997 | 1.00 (0.34-2.98) |
| *PBRM1* | 0.441 | 1.45 (0.56-3.73) | 0.056 | 2.83 (0.97-8.21) |
| **Methylation-related** | 0.094 | 1.69 (0.92-3.10) | 0.506 | 1.29 (0.61-2.69) |
| *KMT2A/C/D* | 0.588 | 1.19 (0.64-2.22) | 0.628 | 1.20 (0.57-2.53) |
| *KMT2A* | 0.667 | 0.77 (0.24-2.51) | 0.687 | 1.37 (0.30-6.27) |
| *KMT2C* | 0.503 | 1.27 (0.64-2.52) | 0.578 | 1.28 (0.54-3.06) |
| *KMT2D* | 0.793 | 0.88 (0.35-2.25) | 0.691 | 1.22 (0.46-3.25) |
| **WNT pathway** | 0.200 | 0.66 (0.36-1.24) | 0.030 | 0.42 (0.20-0.92) |
| *LRP1B* | 0.646 | 0.83 (0.37-1.86) | 0.643 | 0.81 (0.33-1.98) |
| *APC* | 0.660 | 0.77 (0.24-2.49) | 0.108 | 0.36 (0.11-1.25) |
| **Hippo pathway** | 0.057 | 1.81 (0.98-3.32) | 0.925 | 1.04 (0.50-2.13) |
| *FAT1* | 0.336 | 1.78 (0.55-5.80) | 0.742 | 0.77 (0.16-3.71) |
| *FAT3* | 0.071 | 1.86 (0.95-3.64) | 0.703 | 0.85 (0.37-1.94) |
| *NF1/2* | 0.970 | 1.02 (0.40-2.59) | 0.019 | 4.24 (1.27-14.10) |
| **NOTCH pathway** | 0.297 | 1.42 (0.74-2.73) | 0.239 | 1.59 (0.73-3.47) |
| *NOTCH family* | 0.531 | 1.39 (0.49-3.93) | 0.715 | 1.24 (0.39-3.91) |
| *SPEN* | 0.567 | 0.71 (0.22-2.30) | 0.584 | 1.42 (0.41-4.96) |
| *IDH1/2* | 0.489 | 1.65 (0.40-6.87) | 0.273 | 0.42 (0.09-1.99) |

Multivariable Cox model includes mutational event, anatomic site, TNM stage, resection margin, histological grade, and CEA level.

## Supplemental Table S13. Univariable and multivariable analyses of OS in the TCGA cohort.

|  | Univariable analysis | | Multivariable analysis | |
| --- | --- | --- | --- | --- |
| Parameter | HR (95% CI) | P value | HR (95% CI) | P value |
| Age (≥65 vs. <65) | 1.27 (0.50-3.22) | 0.62 |  |  |
| Sex (male vs. female) | 0.72 (0.28-1.84) | 0.49 |  |  |
| Race (white vs. non-white) | 0.47 (0.13-1.72) | 0.25 |  |  |
| Anatomic site (iCCA vs. pCCA+dCCA) | 0.84 (0.24-2.91) | 0.78 | 0.66 (0.07-6.42) | 0.72 |
| Smoking (smoker vs. non-smoker) | 1.42 (0.54-3.77) | 0.48 |  |  |
| TNM stage (III/IV vs. I/II) | 1.48 (0.52-4.21) | 0.47 | 1.04 (0.13-8.28) | 0.97 |
| Resection margin (R0 vs. R1/R2) | 1.57 (0.44-5.65) | 0.49 | 1.18 (0.23-6.16) | 0.85 |
| Histological grade (>G2 vs. ≤G2) | 0.61 (0.23-1.62) | 0.32 | 1.46 (0.25-8.60) | 0.68 |
| Vascular invasion (micro vs. none) | 1.76 (0.49-6.37) | 0.39 |  |  |
| Perineural invasion (yes vs. no) | 4.26 (1.18-15.35) | 0.026 | 9.43 (1.39-64.15) | 0.022 |
| Child-Pugh (B vs. A) | 2.13 (0.25-18.42) | 0.49 |  |  |
| ECOG (≥1 vs. 0) | 1.44 (0.50-4.19) | 0.50 |  |  |
| Cluster-based risk (low vs. high) | 0.47 (0.15-1.46) | 0.19 | 0.25 (0.05-1.31) | 0.100 |

Abbreviations: CCA=cholangiocarcinoma, ECOG=Eastern Cooperative Oncology Group, NA=not applicable, OS=overall survival, TCGA=The Cancer Genome Atlas.

## Supplemental Table S14. Univariable and multivariable analyses of PFI in the TCGA cohort.

|  | Univariable analysis | | Multivariable analysis | |
| --- | --- | --- | --- | --- |
| Parameter | HR (95% CI) | P value | HR (95% CI) | P value |
| Age (≥65 vs. <65) | 0.99 (0.41-2.40) | 0.99 |  |  |
| Sex (male vs. female) | 1.04 (0.42-2.55) | 0.94 |  |  |
| Race (white vs. non-white) | 0.84 (0.24-2.87) | 0.78 |  |  |
| Anatomic site (iCCA vs. pCCA+dCCA) | 2.25 (0.52-9.74) | 0.28 | 3.44 (0.32-37.42) | 0.31 |
| Smoking (smoker vs. non-smoker) | 1.30 (0.52-3.28) | 0.57 |  |  |
| TNM stage (III/IV vs. I/II) | 1.32 (0.48-3.66) | 0.60 | 5.42 (0.93-31.59) | 0.060 |
| Resection margin (R0 vs. R1/R2) | 1.75 (0.58-5.27) | 0.32 | 0.27 (0.05-1.58) | 0.15 |
| Histological grade (>G2 vs. ≤G2) | 0.82 (0.34-1.98) | 0.65 | 0.66 (0.19-2.26) | 0.50 |
| Vascular invasion (micro vs. none) | 1.54 (0.44-5.37) | 0.50 |  |  |
| Perineural invasion (yes vs. no) | 1.64 (0.58-4.60) | 0.35 |  |  |
| Child-Pugh (B vs. A) | 0.91 (0.12-7.12) | 0.93 |  |  |
| ECOG (≥1 vs. 0) | 2.58 (0.97-6.90) | 0.059 | 3.74 (1.00-13.95) | 0.050 |
| Cluster-based risk (low vs. high) | 0.14 (0.04-0.50) | 0.002 | 0.06 (0.01-0.52) | 0.011 |

Abbreviations: CCA=cholangiocarcinoma, ECOG=Eastern Cooperative Oncology Group, NA=not applicable, PFI=progression-free interval, TCGA=The Cancer Genome Atlas.
