# Supplementary material for: Common DNA methylation changes in biliary tract cancers identify subtypes with different immune characteristics and clinical outcomes
Source: BMC Med. 2022 Feb 7;20:64. doi: 10.1186/s12916-021-02197-w (PMC8822710; doi:10.1186/s12916-021-02197-w)
Supplement: Supplementary file 2 — Additional file 2: Method S1. NGS testing for mutation. Method S2. Single sample gene set enrichment analysis (ssGSEA). [file 12916_2021_2197_MOESM2_ESM.docx]

**Additional file 2: Methods S1-2**

Supplement to: B. Li, Z. Qiu, Y. Xu, et al. Common DNA methylation changes of biliary tract cancer identifies subtypes with different immune characteristics and clinical outcomes.

**TABLE OF CONTENTS**

[Supplemental Methods 2](#_Toc87391309)

[Method S1. NGS testing for mutation 2](#_Toc87391310)

[Method S2. Single sample gene set enrichment analysis (ssGSEA) 3](#_Toc87391311)

# Supplemental Methods

## Method S1. NGS testing for mutation

In brief, DNA was fragmented by Covaris M220 focused ultrasonicator (Covaris, Inc., Woburn, MA, USA) followed by end repair, phosphorylation, dA addition, and adaptor ligation for library construction. Then, the DNA library was purified by Agencourt AMPure beads (Beckman Coulter, Fullerton, CA, USA). The quality and the size of the fragments were assessed using Qubit 2.0 fluorimeter with the dsDNA high-sensitivity assay kit (Life Technologies, Carlsbad, CA, USA). Indexed samples were sequenced on Nextseq500 (Illumina, Inc., San Diego, CA, USA) with paired-end reads. Indexed samples were sequenced on Nextseq500 (Illumina, Inc., San Diego, CA, USA) with paired-end reads.

Sequence data were mapped to the human genome (hg19) using BWA aligner 0.7.10. Local alignment optimization, variant calling and annotation were performed using GATK 3.2, MuTect and VarScan. Variants were filtered using the VarScan filter pipeline, with loci with depth less than 100 filtered out. For hotspot insertions and deletions (INDEL), a minimum of 5 supporting reads are needed at an allelic fraction (AF) >.5%; while 10 supporting reads are required for non-hotspot INDEL at an AF >2%. A minimum of 8 supporting reads are needed for hotspot single nucleotide variants (SNV) to be called at an AF>1%, while 16 supporting reads are needed for non-hotspot SNV at an AF >2%. According to the ExAC, 1000 Genomes, dbSNP, ESP6500SI-V2 database, variants with population frequency over 0.1% were grouped as SNP and excluded from further analysis. Remaining variants were annotated with ANNOVAR and SnpEff v3.6. DNA translocation analysis was performed using Factera 1.4.3 as previously described. The limit of detection for SNV is 2% for hotspots and 5% for non-hotspots. Copy number variation was detected by in-house analysis scripts based on depth of coverage data of capture intervals. Coverage data were corrected against sequencing bias resulting from GC content and probe design. The average coverage of all captured regions was used to normalize the coverage of different samples to comparable scales. The copy number was calculated based on the ratio between the depth of coverage in tumor samples and the average coverage of an adequate number (n > 50) of samples without copy number variation as references for each capture interval. Copy number variation was confirmed if the coverage data of the gene region was quantitatively and statistically significantly different from its reference control. The limit of detection for CNVs is 1.5 for deletion and 2.64 for amplification.

## Method S2. Single sample gene set enrichment analysis (ssGSEA)

Based on the transcriptomic data of the TCGA-CHOL cohort, ssGSEA^1 2^ was introduced to estimate the scores concerning the comparisons between naïve, effector, and exhausted CD8^+^ T cells:

1. GSE9650_NAIVE_VS_EFF_CD8_TCELL_UP;
2. GSE9650_NAIVE_VS_EFF_CD8_TCELL_DN;
3. GSE9650_NAIVE_VS_EXHAUSTED_CD8_TCELL_UP;
4. GSE9650_NAIVE_VS_EXHAUSTED_CD8_TCELL_DN;
5. GSE9650_EFFECTOR_VS_EXHAUSTED_CD8_TCELL_UP;
6. GSE9650_EFFECTOR_VS_EXHAUSTED_CD8_TCELL_DN).

We calculated the score of each comparison (e.g., naïve vs. effector) by subtracting the score of DN (downregulation) from the one of UP (upregulation). For instance, a high score of naïve vs. effector may represent that the infiltration level of effector CD8^+^ T cell is higher than that of naïve CD8^+^ T cell.

**References:**

1. Barbie DA, Tamayo P, Boehm JS, et al. Systematic RNA interference reveals that oncogenic KRAS-driven cancers require TBK1. *Nature* 2009;462:108-112.

2. Subramanian A, Tamayo P, Mootha VK, et al. Gene set enrichment analysis: a knowledge-based approach for interpreting genome-wide expression profiles. *Proc Natl Acad Sci U S A* 2005;102:15545-15550.
