# Supplementary material for: Common DNA methylation changes in biliary tract cancers identify subtypes with different immune characteristics and clinical outcomes
Source: BMC Med. 2022 Feb 7;20:64. doi: 10.1186/s12916-021-02197-w (PMC8822710; doi:10.1186/s12916-021-02197-w)
Supplement: Supplementary file 3 — Additional file 3: Fig. S1. Consensus clustering of the EHSH cohort. Consensus matrix of non-supervised clustering of methylation signatures by K-means method (K=2-8) and delta plot assessing change in consensus cumulative distribution function area. Fig. S2. Feature selection using the LASSO algorithm for a prognostic model. A. The optimal tuning parameter (Lambda) in the LASSO model was selected using 3-fold cross-validation. B. LASSO coefficient profiles of the 12 features. Fig. S3. Association between mutational rate and TNM stage. Fig. S4. Consensus clustering of the TCGA-CHOL cohort. Consensus matrix of non-supervised clustering of methylation signatures by K-means method (K=2-8) and delta plot assessing change in consensus cumulative distribution function area. Fig. S5. Overlap of greater infiltration of CD8+ T cell and higher angiogenesis signature clustering in the TCGA-CHOL cohort. Scatter plot illustrating the infiltration of CD8 T cell and the score of angiogenesis signature in the methyl-risk high and the methyl-risk low groups. Fig. S6. Associations of the methyl-risk with the signatures of naïve, effector, and exhausted CD8 T cells in the TCGA-CHOL cohort. Comparisons of the signatures concerning naïve vs. effector, naïve vs. exhausted, and effector vs. exhausted between the two subgroups defined by the methyl-risk in the TCGA-CHOL cohort. [file 12916_2021_2197_MOESM3_ESM.docx]

**Additional file 3: Figures S1-6**

Supplement to: B. Li, Z. Qiu, Y. Xu, et al. Common DNA methylation changes of biliary tract cancer identifies subtypes with different immune characteristics and clinical outcomes.

**TABLE OF CONTENTS**

[Supplemental Figures 2](#_Toc87391412)

[Supplemental Fig. S1. Consensus clustering of the EHSH cohort. 2](#_Toc87391413)

[Supplemental Fig. S2. Feature selection using the LASSO algorithm for a prognostic model. 3](#_Toc87391414)

[Supplemental Fig. S3. Association between mutational rate and TNM stage. 4](#_Toc87391415)

[Supplemental Fig. S4. Consensus clustering of the TCGA-CHOL cohort. 5](#_Toc87391416)

[Supplemental Fig. S5. Overlap of greater infiltration of CD8^+^ T cell and higher angiogenesis signature clustering in the TCGA-CHOL cohort. 6](#_Toc87391417)

[Supplemental Fig. S6. Associations of the methyl-risk with the signatures of naïve, effector, and exhausted CD8 T cells in the TCGA-CHOL cohort. 7](#_Toc87391418)

# Supplemental Figures

## Supplemental Fig. S1. Consensus clustering of the EHSH cohort.


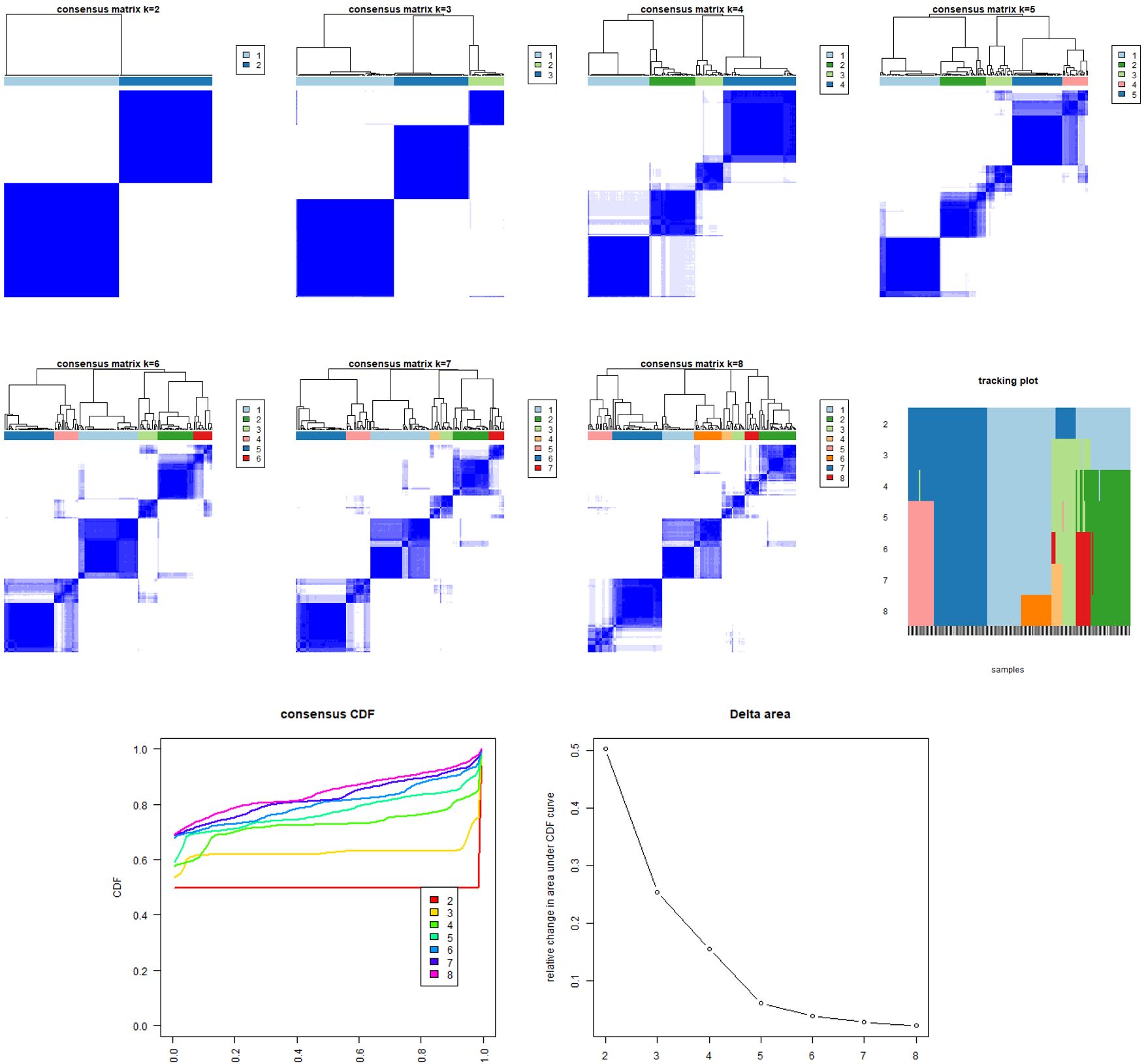


Consensus matrix of non-supervised clustering of methylation signatures by K-means method (K=2-8) and delta plot assessing change in consensus cumulative distribution function area. Abbreviations: CDF=cumulative distribution function.

## Supplemental Fig. S2. Feature selection using the LASSO algorithm for a prognostic model.


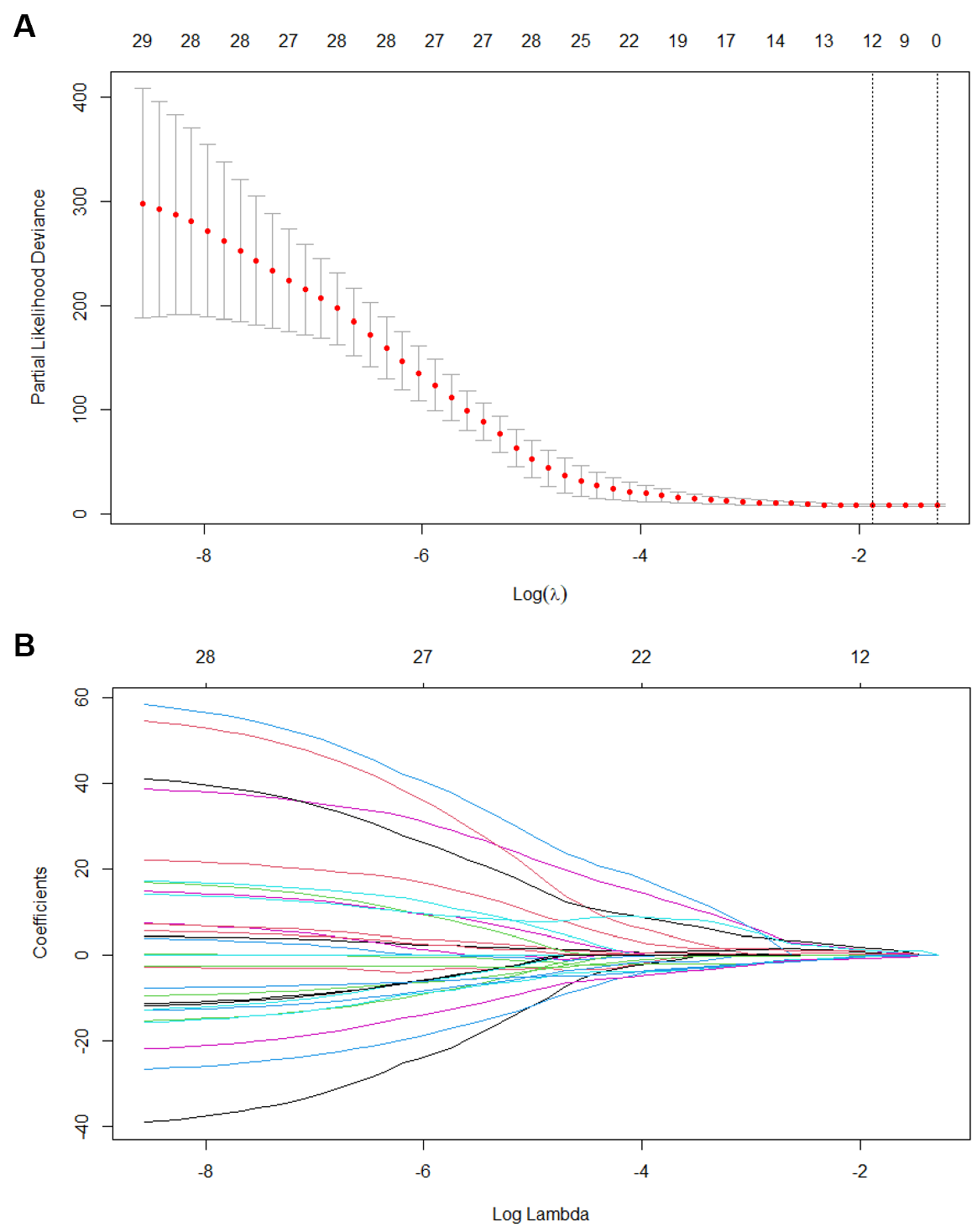


**A**. The optimal tuning parameter (Lambda) in the LASSO model was selected using 3-fold cross-validation. **B**. LASSO coefficient profiles of the 12 features.

## Supplemental Fig. S3. Association between mutational rate and TNM stage.


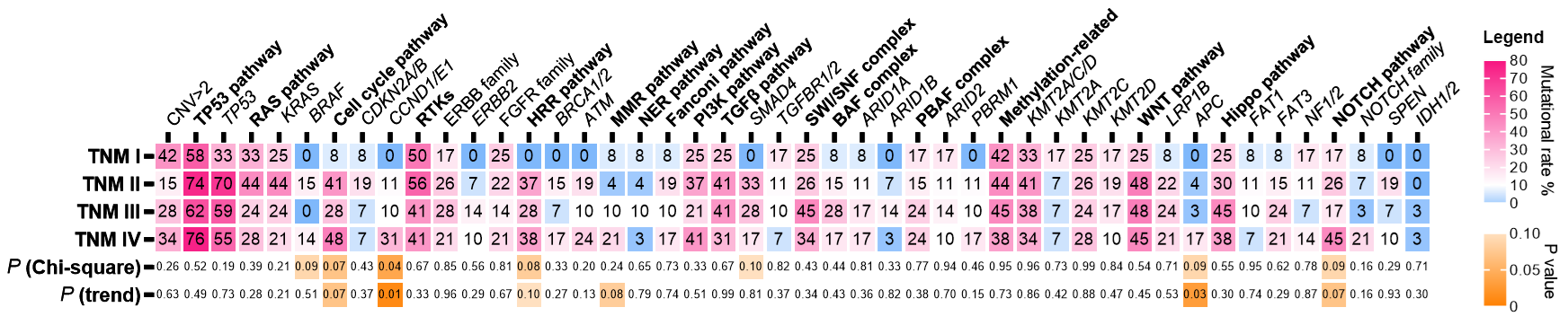


## Supplemental Fig. S4. Consensus clustering of the TCGA-CHOL cohort.


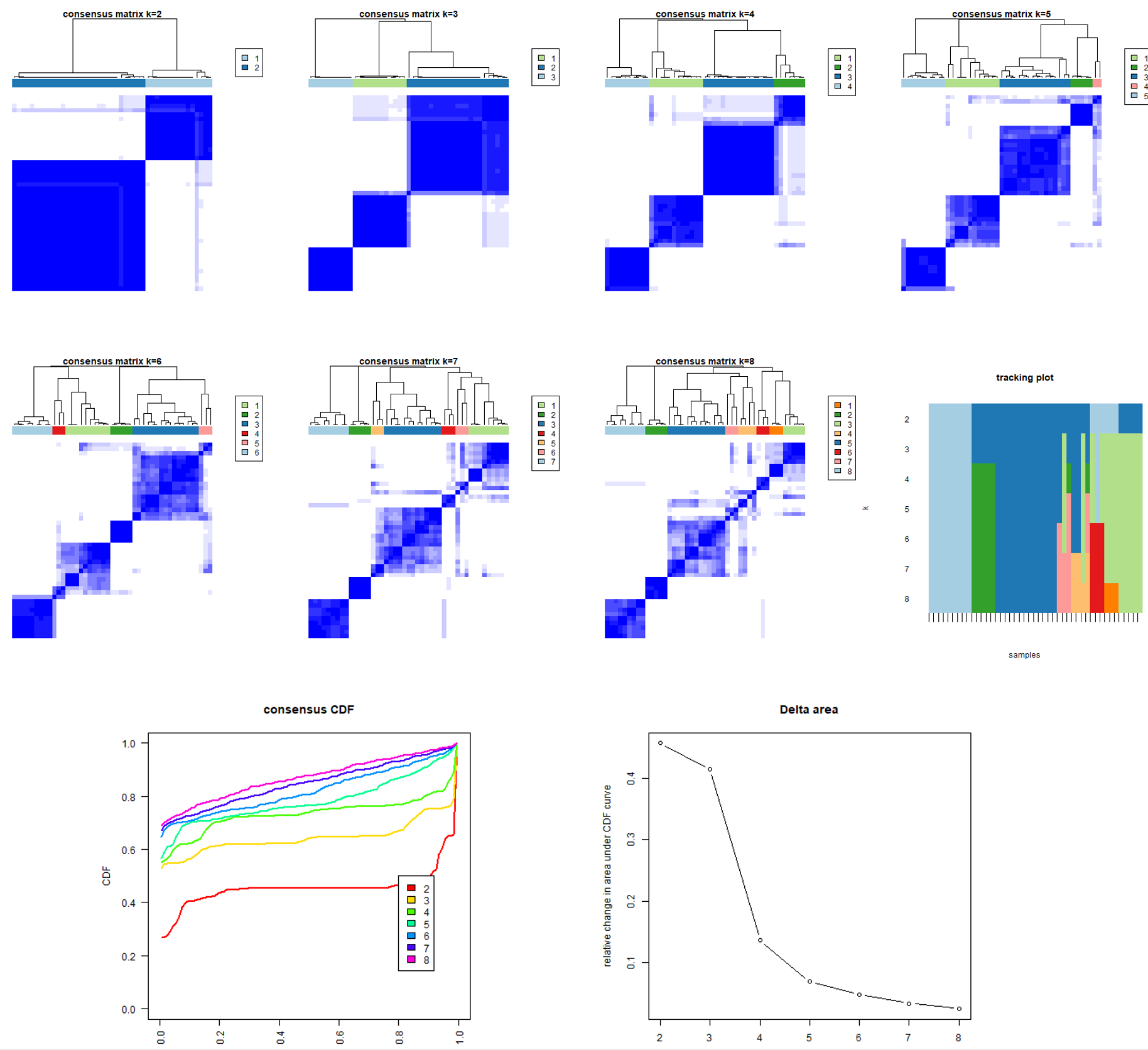


Consensus matrix of non-supervised clustering of methylation signatures by K-means method (K=2-8) and delta plot assessing change in consensus cumulative distribution function area.

## Supplemental Fig. S5. Overlap of greater infiltration of CD8^+^ T cell and higher angiogenesis signature clustering in the TCGA-CHOL cohort.


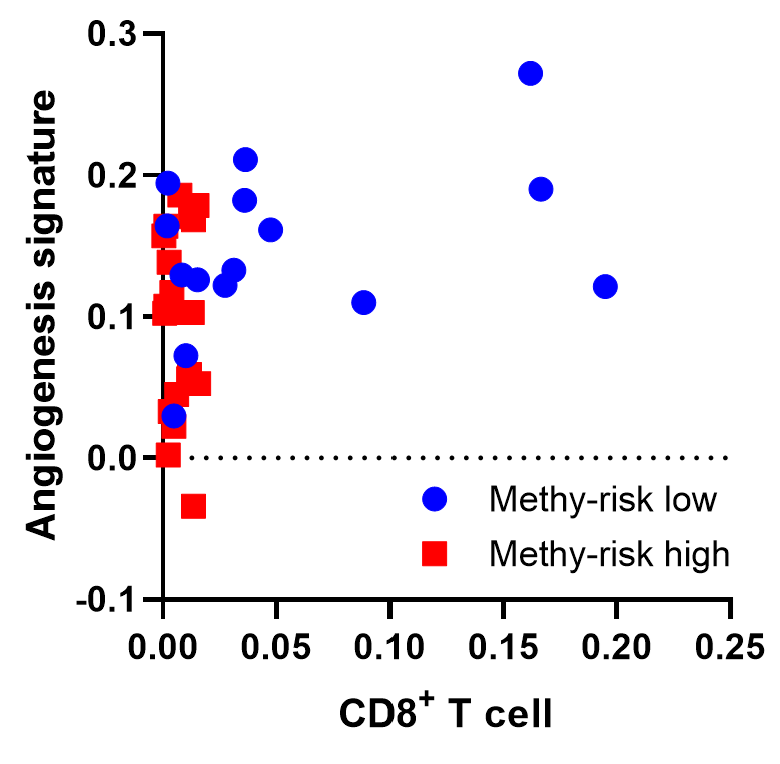


Scatter plot illustrating the infiltration of CD8 T cell and the score of angiogenesis signature in the methyl-risk high and the methyl-risk low groups.

## Supplemental Fig. S6. Associations of the methyl-risk with the signatures of naïve, effector, and exhausted CD8 T cells in the TCGA-CHOL cohort.


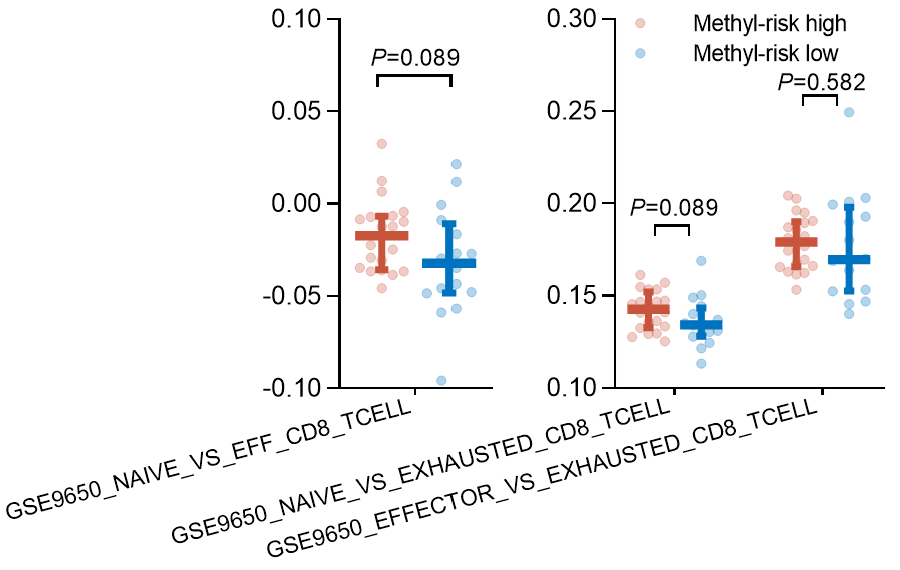


Comparisons of the signatures concerning naïve vs. effector, naïve vs. exhausted, and effector vs. exhausted between the two subgroups defined by the methyl-risk in the TCGA-CHOL cohort.
